# A polysaccharide deacetylase enhances bacterial adhesion in high-ionic-strength environments

**DOI:** 10.1016/j.isci.2021.103071

**Published:** 2021-09-02

**Authors:** Nelson K. Chepkwony, Yves V. Brun

**Affiliations:** 1 Département de microbiologie, infectiologie et immunologie, Université de Montréal, C.P. 6128, succ. Centre-ville, Montréal, QC H3C 3J7, Canada

**Keywords:** Biochemistry, Microbiology, Microbiofilms

## Abstract

Differences in ionic strength, pH, temperature, shear forces, and other environmental factors impact adhesion, and organisms have evolved various strategies to optimize their adhesins for their specific environmental conditions. Many species of Alphaproteobacteria, including members of the order Caulobacterales, use a polar adhesin, called holdfast, for surface attachment and subsequent biofilm formation in both freshwater and marine environments. *Hirschia baltica,* a marine member of Caulobacterales, produces a holdfast adhesin that tolerates a drastically higher ionic strength than the holdfast produced by its freshwater relative, *Caulobacter crescentus*. In this work, we show that the holdfast polysaccharide deacetylase HfsH plays an important role in adherence in high-ionic-strength environments. We show that increasing expression of HfsH improves holdfast binding in high-ionic-strength environments. We conclude that HfsH plays a role in modulating holdfast binding at high ionic strength and hypothesize that this modulation occurs through varied deacetylation of holdfast polysaccharides.

## Highlights

• The polysaccharide deacetylase HfsH is required for *H. baltica* adhesion
• Holdfast polysaccharides in *H. baltica* Δ*hfsH* lack cohesive and adhesive properties
• HfsH expression correlates positively with holdfast binding in high ionic strength
• HfsH is an important factor for adherence in high-ionic-strength environments

## Graphical abstract

**Figure:**
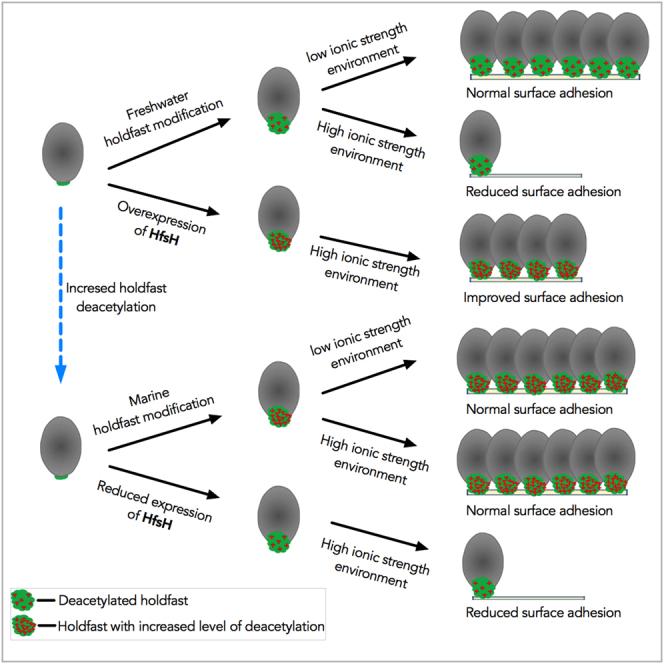

## Introduction

The development of adhesives that perform well on wet surfaces has been a challenge for centuries, yet this problem has been solved multiple times during the evolution of sessile aquatic organisms. These organisms derive multiple benefits from their adhesion to surfaces in aquatic environments such as increased access to nutrients, aerated water, and protection from predation. Aquatic environments can differ in ionic strength, pH, temperature, and shear forces, requiring the evolution of environment-optimized adhesion strategies. For example, mussels, a diverse group of bivalve mollusk species, can attach to surfaces in freshwater, brackish waters, and marine habitats, suggesting a successful evolution of adhesion mechanisms adapted to different ionic environments (Maier et al., 2015; Waite, 2017). Both marine and freshwater mussels produce a fibrous polymeric adhesin structure called the byssus for surface attachment (Maier et al., 2015; Waite, 2017). Mussel byssus-mediated adhesion is one of the best characterized systems for how adhesins interact with wet surfaces in both low- and high-ionic-strength environments (Waite, 2017; Lee et al., 2011). Despite the impressive progress in understanding the mechanistic basis for mussel adhesion in different-ionic-strength environments, the lack of a genetic system has made it difficult to study the evolution of those mechanisms.

Here, we use genetically tractable, related freshwater and marine species of the order Caulobacterales to investigate the evolution of adhesion in these two environments. Most bacteria spend their lives attached to or associated with surfaces. Bacteria attach to surfaces using adhesins, which are mainly composed of polysaccharides, DNA, and/or proteins (Berne et al., 2015, 2018). The mechanism by which adhesins interact with different surfaces is still unclear, but studies have shown that electrostatic interactions play an important role (Chen et al., 2011; Tuson and Weibel, 2013, Ruffatto et al., 2014; Garrels and Thompson, 1962). In marine environments, bacterial adhesins face high ionic strengths, up to 600 mM, compared with ∼0.05 mM in freshwater lakes and ponds (Garrels and Thompson, 1962; Zita and Hermansson, 1994). Nevertheless, marine bacteria attach efficiently to surfaces in the ocean, despite shielding of electrostatic forces that contribute to surface adhesion in high-ionic-strength environments (Chen and Walker, 2007, De Carvalho, 2018). Therefore, bacteria living in such environments must use adhesins that are adapted to binding at high ionic strength.

**Figure 1:**
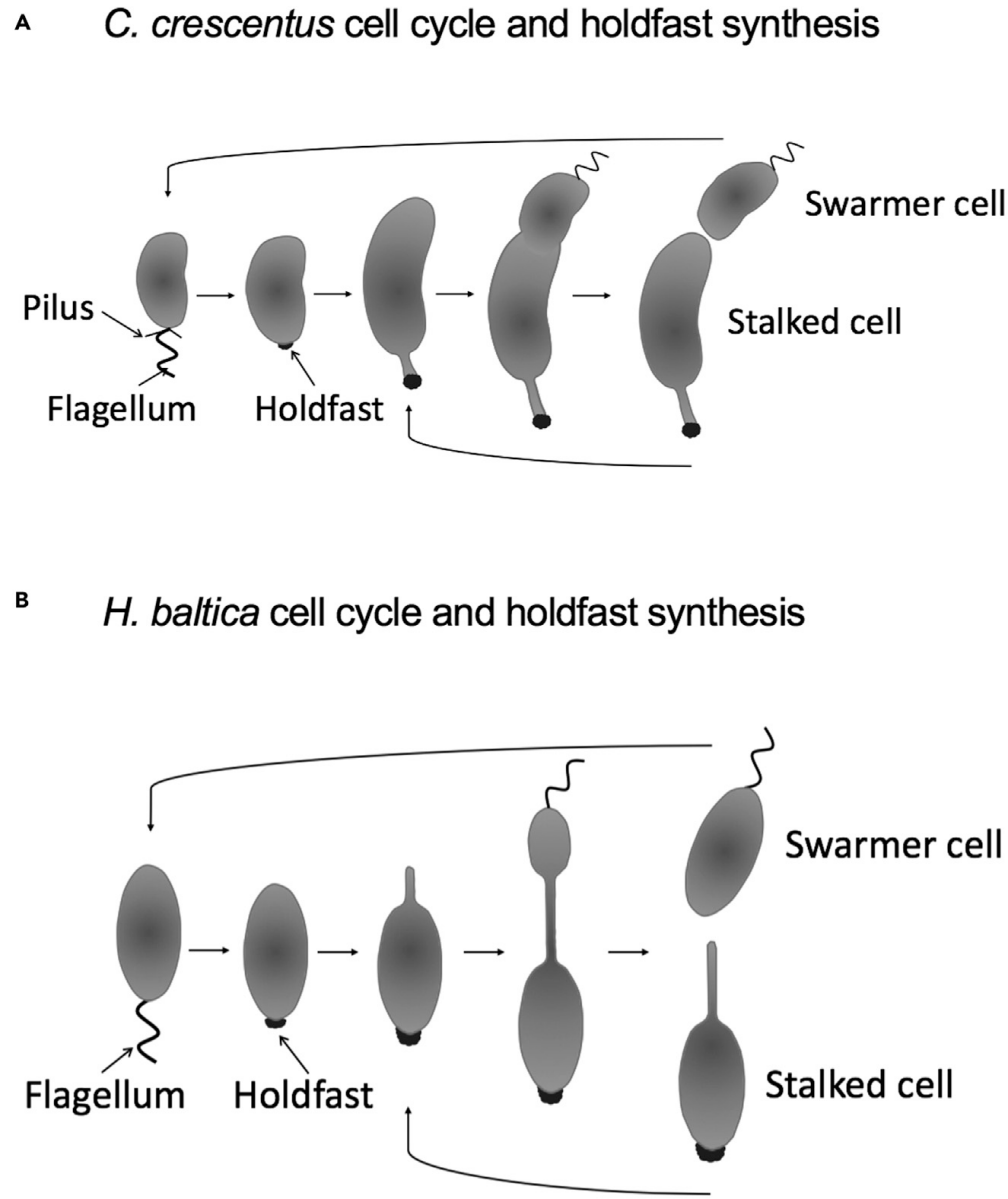
Cell cycle and holdfast synthesis of *C. crescentus* and *H. baltica* (A) Diagram of the *C. crescentus* dimorphic cell cycle. A motile swarmer cell differentiates into a stalked cell by shedding the flagellum, retracting the pili, and synthesizing a holdfast-tipped stalk at the same cell pole. *C. crescentus* stalked cells divide asymmetrically to produce a motile swarmer cell and a surface-adherent stalked cell. (B) Diagram of the *H. baltica* dimorphic cell cycle. A motile swarmer cell differentiates into a stalked cell by shedding its flagellum and synthesizing holdfast at the same cell pole. At the opposite pole, a budding stalk is synthesized that is used to bud a new motile swarmer cell.

Species in the order Caulobacterales are found as surface-attached cells growing in a diverse environment. Their natural habitat ranges from freshwater and marine aquatic environments to nutrient-rich soil and the rhizosphere (Wilhelm, 2018; Poindexter, 1964). Cells attach permanently to surfaces using a specialized polar adhesin called holdfast (Merker and Smit, 1988; Poindexter, 1964). *Caulobacter crescentus*, a freshwater member of the Caulobacterales*,* is a stalked bacterium with a dimorphic cell cycle that fluctuates between a flagellated, motile swarmer and a sessile stalked cell (Poindexter, 1964). A swarmer cell differentiates into a stalked cell by shedding its flagellum and synthesizing holdfast-tipped stalk at the same pole (Figure 1A). Although the exact composition of the *C. crescentus* holdfast is unknown, it has been shown to contain the monosaccharides *N*-acetylglucosamine (GlcNAc), glucose, 3-*O*-methylglucose, mannose, and xylose (Merker and Smit, 1988; Hershey et al., 2019), as well as proteins and DNA (Hernando-Pérez et al., 2018). The *C. crescentus* holdfast attaches to surfaces with a strong adhesive force of 70 N/mm^2^ (Berne et al., 2013; Tsang et al., 2006).

Caulobacterales use similar genes to synthesize, export, and anchor the holdfast (Berne et al., 2015; Chepkwony et al., 2019), yet there are substantial differences in holdfast binding properties at high ionic strength. Most studies of holdfast properties have been performed in the freshwater *C. crescentus* (Berne et al., 2018), in which as little as 10 mM NaCl leads to a 50% reduction in binding to glass (Berne et al., 2013). Recently, we studied a marine Caulobacterales *Hirschia baltica,* which produces holdfast at the cell pole and uses the stalk for budding as shown on Figure 1B (Chepkwony et al., 2019). *H. baltica* produces holdfasts that tolerate a significantly higher ionic strength than *C. crescentus* holdfast, where 600 mM NaCl was required to observe a 50% decrease in binding to glass (Chepkwony et al., 2019). Differences in holdfast tolerance of ionic strength could result from differences in molecular composition or the degree or type of modification.

Two holdfast modifying enzymes that have been characterized in the freshwater *C. crescentus* are the putative acetyltransferase, HfsK (Sprecher et al., 2017), and the putative polysaccharide deacetylase, HfsH (Wan et al., 2013). Deletion of *hfsK* or *hfsH* reduces holdfast cohesiveness (holdfast intramolecular interactions) and adhesiveness (holdfast-surface interactions), which leads to shedding of holdfasts into the medium (Sprecher et al., 2017; Wan et al., 2013). This shedding phenotype is similar to that observed in mutants lacking holdfast anchor proteins (Cole et al., 2003; Hardy et al., 2010). Furthermore, overexpression of HfsH in *C. crescentus* increases cell adhesion without increasing the amount of holdfast produced (Wan et al., 2013), implying that not all sugar subunits are deacetylated in wild-type (WT) holdfast. Interestingly, studies on deacetylation of the GlcNAc polymer chitin indicate that removal of acetyl groups leaves the resultant chitosan with an exposed amine group (Sorlier et al., 2001). The level of deacetylation of chitosan changes its physical and chemical properties by altering electrostatic interactions, acid-base interactions, hydrogen bonds, and hydrophobic interactions with surfaces (Sorlier et al., 2001). Therefore, we hypothesized that the partial positive charge on the primary amine formed after deacetylation of the holdfast GlcNAc polysaccharide by HfsK and/or HfsH might play a role in improving holdfast binding in high-ionic-strength environments.

In the present study, we show that the polysaccharide deacetylase HfsH is required for *H. baltica* adhesion and holdfast binding. We demonstrate that holdfast produced by a *H. baltica* Δ*hfsH* mutant is deficient in both cohesive and adhesive properties. *H. baltica* Δ*hfsH* produces a similar quantity of holdfast polysaccharide as the WT one, but owing to a lack of cohesiveness and adhesiveness, these holdfasts disperse into the medium. Furthermore, we demonstrate that holdfast binding can be modulated by varying the level of expression of HfsH. In *C. crescentus*, overexpression of HfsH increases ionic strength tolerance of holdfasts, while reducing expression of HfsH in *H. baltica* results in reduced ionic strength tolerance. Finally, we show that *H. baltica* HfsH helps to maintain the integrity of the holdfast structure, as holdfasts produced by a *H. baltica* Δ*hfsH* mutant lose their protein and galactose constituents. Collectively our results suggest that modulation of the level of the holdfast polysaccharide deacetylase HfsH is an important adaptation for adherence in high-ionic-strength environments.

## Results

### The holdfast polysaccharide deacetylase HfsH is required for adhesion and biofilm formation in *H. baltica*

**Figure 2:**
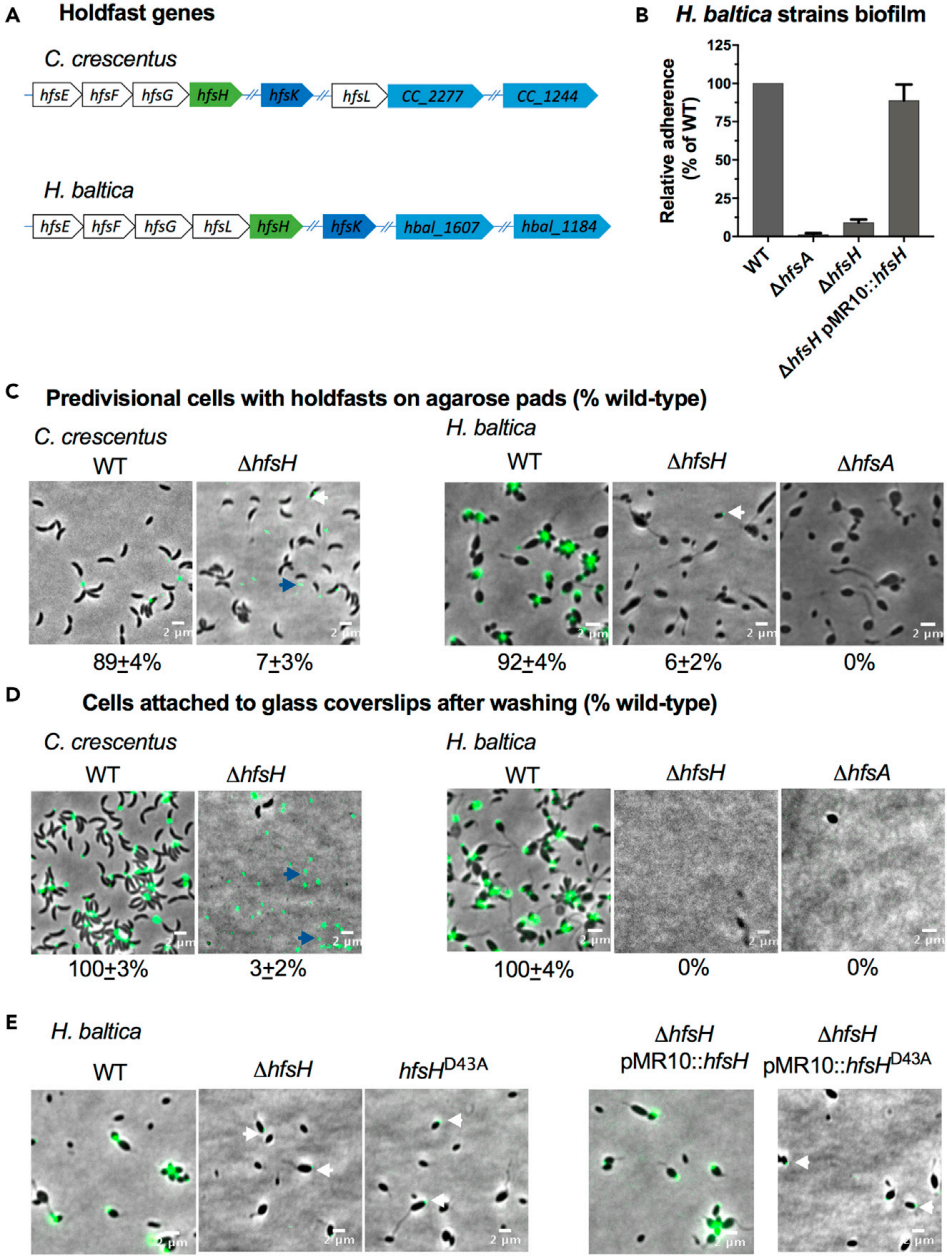
The role of HfsH and HfsK in *H. baltica* holdfast biogenesis (A) Genomic organization of holdfast synthesis (*hfs)* genes in *C. crescentus* and *H. baltica.* Genes were identified using reciprocal best hit analysis on *C. crescentus* and *H. baltica* genomes. In both *C. crescentus* and *H. baltica* genomes*, hfsH* is found in the *hfs* locus while *hfsK* and its paralogs are found outside the *hfs* locus. Color coding corresponds to homologs and paralogs. Hash marks indicate genes that are found in a different location in the genome. (B) Quantification of biofilm formation by the crystal violet assay after incubation for 12 h, expressed as a mean percent of WT crystal violet staining. Holdfast null strain *ΔhfsA* was used as a negative control. Error is expressed as the standard error of the mean of three independent biological replicates with four technical replicates each. (C) Representative images showing merged phase and fluorescence channels of the indicated *C. crescentus* and *H. baltica* strains on agarose pads. Holdfast is labeled with AF488-WGA (green). White arrows indicate holdfasts attached to the *ΔhfsH* cells, and blue arrows indicate holdfast shed into the medium. Scale bar, 2 μm. Exponential planktonic cultures were used to quantify the percentage of predivisional cells with holdfast. Data are expressed as the mean of three independent biological replicates with four technical replicates each. Error bars represent the standard error of the mean. A total of 3,000 cells were quantified per replicate using MicrobeJ. (D) Representative images showing merged phase and fluorescence channels of *C. crescentus* and *H. baltica* strains bound to a glass coverslip. Exponential cultures were incubated on the glass slides for 1 h and washed to remove unbound cells, and holdfasts were labelled with AF488-WGA (green). Blue arrows indicate surface-bound holdfasts shed by *hfsH* mutants. Scale bar, 2 μm. The data showing quantification of cells bound to the glass coverslip are the mean of two biological replicates with five technical replicates each. Error is expressed as the standard error of the mean using MicrobeJ. (E) Representative images showing merged phase and fluorescence channels of *H. baltica* strains with holdfast polysaccharides labeled with AF488-WGA (green) on agarose pads. Scale bar, 2 μm. A point mutation was introduced at a key substrate binding residue in *H. baltica* HfsH, resulting in an amino acid change from aspartic acid to alanine at position 43 (D43A). White arrows indicate faint AF488-WGA holdfast labeling on mutant cells

A putative acetyltransferase HfsK and a polysaccharide deacetylase HfsH modulate *C. crescentus* holdfast binding properties (Sprecher et al., 2017; Wan et al., 2013). In *C. crescentus*, deacetylation of holdfast polysaccharides is important for both the cohesiveness and adhesiveness of holdfasts (Wan et al., 2013). In our previous work comparing *H. baltica* and *C. crescentus* holdfasts (Chepkwony et al., 2019), we showed that both species use similar genes to synthesize, export, and anchor holdfasts to the cell envelope. We identified *H. baltica* genes that modify holdfast in *C. crescentus*, namely the putative acetyltransferase *hfsK* (*hbal_0069)* and the polysaccharide deacetylase *hfsH* (*hbal_1965*; Figure 2A). In *C. crescentus,* the *hfsK* gene as well as its paralogs *CC_2277* and *CC_1244* are found outside the core *hfs* locus (Figure 2A). Similar to *C. crescentus,* the *H. baltica hfsK* gene and its paralogs *hbal_1607* and *hbal_1184* are also found outside the *hfs* locus (Figure 2A). Basic local alignment search tool (BLAST) analysis did not identify any additional *hfsK* paralogs in *H. baltica*.

*C. crescentus* HfsK is involved in holdfast modification, although its role is unclear (Sprecher et al., 2017). *C*. *crescentus* Δ*hfsK* produces holdfasts that are less adhesive, are not cohesive, and are shed into the medium (Sprecher et al., 2017). In a glass surface binding assay, *C*. *crescentus* Δ*hfsK* produces holdfasts that adhere to glass but fail to anchor cells in place (Sprecher et al., 2017). To test whether *hfsK* and its paralogs play a role in biofilm formation in *H. baltica,* we generated in-frame deletion mutants of *H. baltica hfsK* and its paralogs *hbal_1607* and *hbal_1184*. The *H. baltica ΔhfsK* mutant showed no defect in biofilm formation after 12 h of incubation at room temperature (Figure S1A). We observed similar results for the *H. baltica* Δ*hbal_1607* and the *H. baltica* Δ*hbal_1184* mutants, as well as the triple deletion mutant *H. baltica ΔhfsK Δhbal_1607 Δhbal_1184* (Figure S1A). These results indicate that HfsK and its paralogs are not involved *H. baltica* biofilm formation, in contrast to what has been reported for *C. crescentus* (Sprecher et al., 2017) and *H. baltica hfsH* mutant (Figure S1A).

As holdfast is required for biofilm formation in *C. crescentus* and *H. baltica* (Chepkwony et al., 2019; Ong et al., 1990; Merker and Smit, 1988)*,* we probed for the presence of holdfasts using fluorescent Alexa Fluor 488 (AF488) conjugated wheat germ agglutinin (WGA) lectin that specifically binds to the GlcNAc component of the holdfast polysaccharide (Merker and Smit, 1988). In exponentially growing planktonic cultures, *C. crescentus* WT cells produced holdfasts that bound AF488-WGA and formed cell-cell aggregates mediated by holdfasts, called rosettes (Figure S1B, left panel). *C. crescentus ΔhfsK* produced holdfasts that variably were associated with the cell or were shed into the medium (Figure S1B, left panel, white and blue arrows), as previously shown (Sprecher et al., 2017). Deletion of *hfsK* in *H. baltica* had no effect on AF488-WGA binding to holdfast, rosette formation, or holdfast shedding (Figure S1B), consistent with its lack of an effect on biofilm formation (Figure S1A).

To test whether *H. baltica* HfsK is involved in holdfast anchoring, we spotted exponentially growing cultures on a glass coverslip and incubated for 1 h at room temperature to allow for binding to the coverslip. Unbound cells were removed by washing, and AF488-WGA was added to label holdfasts that remained attached to the coverslip. As a control, *C. crescentus* and *H. baltica* WT cells were incubated with coverslips, and adherent holdfasts were labeled with AF488-WGA (Figure S1C). *C. crescentus ΔhfsK* holdfasts were bound to coverslips but appeared to be spread over the surface, covering a greater area than WT and suggesting that they may be less cohesive (Figure S1C), in agreement with previous studies (Sprecher et al., 2017). These holdfasts also failed to anchor *C. crescentus ΔhfsK* cells to the surface (3% of WT, Figure S1C). In comparison, mutants with deletion of *hfsK* and its paralogs in *H. baltica* produced holdfasts that were bound to the glass surface and formed rosettes similarly to WT (Figure S1C right panel). Interestingly, deletion of the *H. baltica hfsK* paralog *hbal*_*1184* led to the generation of large cellular aggregates that formed independently of holdfast biogenesis (Figure S1D). These cells had morphological defects and were surrounded by debris that may have resulted from cell lysis, indicating that Hbal_1184 is likely involved in a different polysaccharide biosynthetic pathway that contributes to cellular viability. We conclude that HfsK and its paralogs do not contribute to *H. baltica* holdfast-binding properties under our assay conditions (Figures S1A–S1C).

Next, we examined the role of the polysaccharide deacetylase HfsH (Hbal_1965) in *H. baltica* biofilm formation by generating an in-frame deletion mutant of *hfsH*. *H. baltica ΔhfsH* was deficient in biofilm formation, similarly to a holdfast null strain *ΔhfsA* (Figure 2B)*,* and this phenotype could be restored by complementation in *trans* by a replicating plasmid encoding a copy of the *H. baltica hfsH* gene under the control of its native promoter (Figure 2B). These results show that the polysaccharide deacetylase HfsH plays a significant role in biofilm formation in *H. baltica.*

As holdfast is required for biofilm formation in *C. crescentus* and *H. baltica* (Chepkwony et al., 2019; Ong et al., 1990; Merker and Smit, 1988), we probed for the presence of holdfasts using AF488-WGA lectin that specifically binds to the GlcNAc component of the holdfast polysaccharide. In exponentially growing planktonic cultures, *C. crescentus* WT cells produced holdfasts that bound AF488-WGA and formed cell-cell aggregates mediated by holdfasts, called rosettes (Figure 2C, left panel). *C. crescentus* holdfast is produced at the tip of the stalk, a thin extension of the cell body (Figure 1A). *C. crescentus ΔhfsH* produced holdfasts which were associated with the cell or were shed into the medium, as previously shown (Wan et al., 2013). In planktonic culture, *H. baltica* WT formed rosettes and produced holdfasts that bound AF488-WGA and are associated to the cells (Figure 2C, right panel). *H. baltica* Δ*hfsH* did not form rosettes, and only 6% of the cells showed some AF488-WGA staining, while many had no AF488-WGA staining similarly to holdfast null mutant Δ*hfsA*, suggesting that no holdfast was associated with these cells (Figure 2C, right panel, white arrows). Furthermore*,* we did not observe shed holdfast in the medium from *H. baltica ΔhfsH* (Figure 2C, right panels), which contrasts with the *C. crescentus ΔhfsH* phenotype (Figure 2C, left panels, white and blue arrows). Holdfast and rosette formation could be restored in the Δ*hfsH* mutants by complementing in *trans* with a replicating plasmid encoding a copy of the *hfsH* gene (Figure S2A). We observed that *C. crescentus ΔhfsH* produced small holdfasts which bound to coverslips (Figure 2D, left panels, blue arrows and Figure S2B) but failed to anchor the cells (3% of WT, Figure 2D), as previously show (Wan et al., 2013). In contrast, *H. baltica* Δ*hfsH* cells did not bind to the coverslip and we could not detect AF488-WGA labeling on the coverslip surface similarly to a holdfast null mutant Δ*hfsA* (Figure 2D, right panel), suggesting that holdfasts failed to attach to the glass surface.

The genomes of freshwater and marine Caulobacterales have a conserved *hfsH* gene in the core holdfast synthesis locus (Chepkwony et al., 2019). HfsH is a predicted carbohydrate esterase family 4 (CE4) enzyme (Wan et al., 2013). The CE4 family of polysaccharide deacetylases have five catalytic motifs for substrate and cofactor binding, as well as those that participate directly in the catalytic mechanism (Tuveng et al., 2017), which are all present in *C. crescentus* HfsH (HfsH_CC_) and in *H. baltica* HfsH (HfsH_HB_, Figure S2C) . To test if *H. baltica* HfsH is a holdfast polysaccharide deacetylase, we engineered a point mutation in a key substrate-binding residue, resulting in an amino acid change from aspartic acid to alanine at position 43 (D43A; Figure S2C, asterisk). We monitored for the presence of holdfast using fluorescence microscopy with AF488-WGA. Introduction of D43A in the *H. baltica hfsH* gene (*hfsH*_*HB*_^*D43A*^) phenocopied the *hfsH* deletion (Figure 2E, white arrows). We complemented the *H. baltica ΔhfsH* and *C. crescentus ΔhfsH* mutants with a WT copy of *H. baltica hfsH*_*HB*_*,* or with the point mutant *hfsH*_*HB*_^*D43A*^, expressed under their respective native holdfast synthesis locus promoters on a low copy replicating plasmid (pMR10). Although WT *hfsH*_*HB*_ and active site mutant *hfsH*_*HB*_^*D43A*^ were expressed similarly (Figure S2D), complementation with the WT allele restored AF488-WGA holdfast labeling in both *H. baltica ΔhfsH* and *C. crescentus ΔhfsH* mutants, while the point mutant *hfsH*_*HB*_^*D43A*^ did not (Figures 2E and S2E). Because we had showed in previous work that *C. crescentus* HfsH has deacetylase activity that is abolished by the equivalent mutation, these data provide strong genetic support for *H. baltica* HfsH deacetylase activity (Wan et al., 2013). These results confirm that *H. baltica* HfsH is involved in holdfast biogenesis and that D43 is important for its activity, similarly to *C. crescentus* HfsH (Wan et al., 2013).

The aforementioned results indicate that HfsH plays an important role in *H. baltica* holdfast properties, including their anchoring to the cell envelope. We hypothesized that (1) *H. baltica* Δ*hfsH* produces a small amount of holdfast polysaccharide that is insufficient to anchor the cell to the surface, similarly to the underexpression of a glycosyltransferase HfsL (Chepkwony et al., 2019), or (2) holdfasts produced by *H. baltica* Δ*hfsH* are deficient in adhesion and/or cohesion and are thus dispersed into the medium.

### Holdfast produced by *H. baltica ΔhfsH* forms thread-like fibers that diffuse into the medium

**Figure 3:**
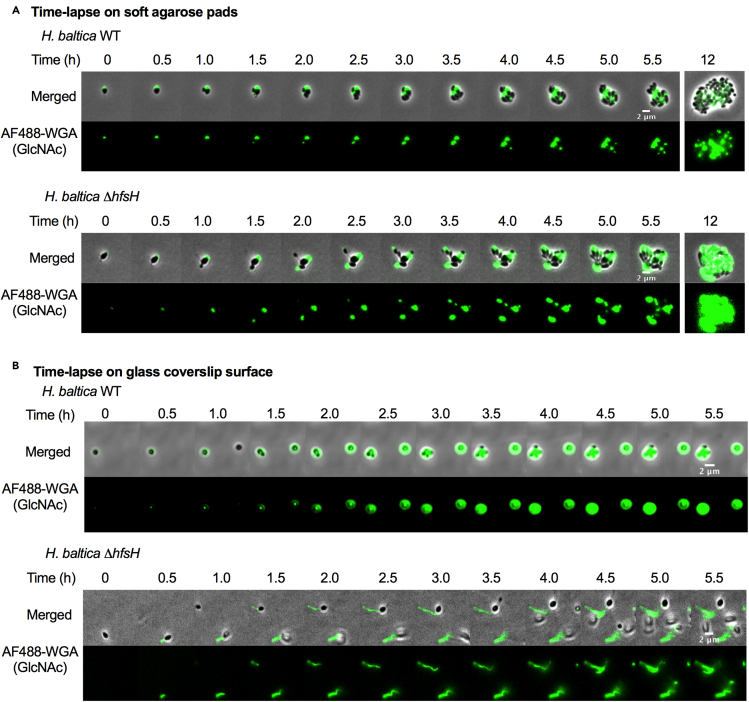
*H. baltica hfsH* mutant holdfasts forms thread-like fibers that diffuse into the medium (A) Time-lapse montages of *H. baltica* WT and *H. baltica ΔhfsH* on soft agarose pads. Exponential cultures were placed on soft agarose pads containing holdfast-specific AF488-WGA (green) and covered with a glass coverslip. Images were collected every 5 min for 12 h. Scale bar, 2 μm. (B) Time-lapse montages of *H. baltica* WT and *H. baltica ΔhfsH* in microfluidic channels. Exponential cultures with holdfast-specific AF488-WGA (green) were injected into the microfluidic chambers, and flow was turned off. Images were collected every 20 s for 5.5 h. Scale bar, 2 μm. See also Videos S1, S2, S3, and S4.

The deacetylase mutant *H. baltica* Δ*hfsH* showed a more severe holdfast attachment deficiency than *C. crescentus* Δ*hfsH (*Figures 2C and 2D*).* Although we did not observe any holdfast binding to glass coverslips for the *H. baltica* Δ*hfsH* mutant, cells grown planktonically had a faint AF488-WGA labeling (Figure 2C). These results prompted us to perform time-lapse microscopy to better understand production of holdfast by *H. baltica ΔhfsH*. To visualize holdfast production, we spotted exponential-phase cells on top of a soft agarose pad containing AF488-WGA and collected images every 5 min for 12 h. We observed that *H. baltica* WT produced holdfasts that were labelled with AF488-WGA (Figure 3A, upper panels) and that the *ΔhfsH* mutant initially produced holdfasts similarly to WT (Figure 3A, lower panels). However, the holdfasts produced by *H. baltica ΔhfsH* appeared more diffuse than WT over time (Figure 3A; Videos S1 and S2). These results show that *H. baltica ΔhfsH* produces holdfast material, indicating that HfsH is not essential for holdfast synthesis.

Video S1. *H. baltica hfsH* mutant holdfasts forms thread-like fibers that diffuse into the medium, related to Figure 3 Time-lapse video of *H. baltica* WT on soft agarose pads. Exponential cultures were placed on soft agarose pads containing holdfast-specific AF488-WGA (green) and covered with a glass coverslip. Images were collected every 5 min for 12 h.

Video S2. *H. baltica hfsH* mutant holdfasts forms thread-like fibers that diffuse into the medium, related to Figure 3 Time-lapse video of *H. baltica ΔhfsH* on soft agarose pads. Exponential cultures were placed on soft agarose pads containing holdfast-specific AF488-WGA (green) and covered with a glass coverslip. Images were collected every 5 min for 12 h.

To test how holdfast produced by *H. baltica ΔhfsH* interacts with a glass surface, we performed time-lapse microscopy using a microfluidic device with a low flow rate (1.4 μL/min). We injected exponential-phase cells mixed with AF488-WGA into the microfluidic chamber, turned off the flow, and imaged the cells every 20 s for 5.5 h. In the microfluidic chamber, we observed that *H. baltica* WT cells arrived at the glass surface and produced holdfasts, allowing them to remain bound to the surface (Figure 3B, upper panels and Video S3). In contrast*, H. baltica ΔhfsH* produced holdfasts that did not remain cohesive on the glass surface and instead formed thread-like fibers (Figure 3B, lower panel and Video S4). These results indicate that HfsH in *H. baltica* is not required for holdfast synthesis but is essential for maintenance of holdfast cohesive properties.

Video S3. *H. baltica hfsH* mutant holdfasts forms thread-like fibers that diffuse into the medium, related to Figure 3 Time-lapse video of *H. baltica* WT in microfluidic channels. Exponential cultures with holdfast-specific AF488-WGA (green) were injected into the microfluidic chambers and flow was turned off. Images were collected every 20 sec for 5.5 h.

Video S4. *H. baltica hfsH* mutant holdfasts forms thread-like fibers that diffuse into the medium, related to Figure 3 Time-lapse video of *H. baltica ΔhfsH* in microfluidic channels. Exponential cultures with holdfast-specific AF488-WGA (green) were injected into the microfluidic chambers and flow was turned off. Images were collected every 20 sec for 5.5 h.

### HfsH expression correlates with the level of biofilm formation

**Figure 4:**
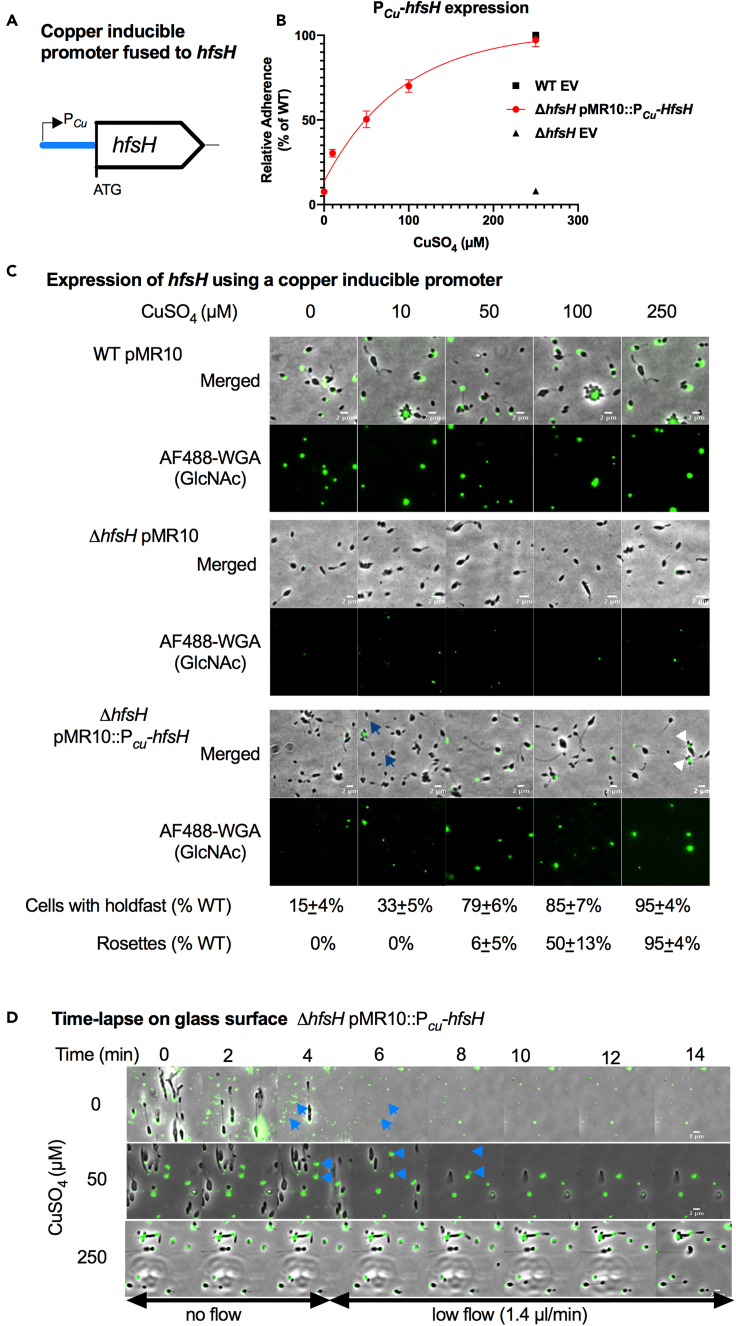
HfsH expression correlates to the level of biofilm formation (A) Schematic representation of *hfsH* under the control of a copper-inducible promoter. 500-bp upstream of the *copA* open reading frame corresponding to the promoter region, P_*Cu*_, were fused to *hfsH* from *H. baltica* and assembled into the plasmid pMR10. (B) A nonlinear regression plot showing quantification of adhesion of *H. baltica* strains induced with 0–250 μM of CuSO_4_ for 4 h by the crystal violet assay. Data are expressed as a mean percent of WT crystal violet staining from three independent biological replicates with four technical replicates. Error is expressed as the standard error of the mean. EV is empty vector (pMR10). (C) Representative images of *H. baltica* WT*, H. baltica ΔhfsH*, and *H. baltica ΔhfsH* complemented with pMR10:P_*Cu*_*-hfsH*. Holdfasts were labeled with AF488-WGA (green). Exponential cultures were induced for 2 h with 0–250 μM of CuSO_4_. Blue arrows indicate shed holdfast at low levels of induction (10 μM CuSO_4_), and white arrowheads indicate rosettes formed at high levels of HfsH induction (250 μM CuSO_4_). Scale bar, 2 μm. (D) Time-lapse montages of *H. baltica ΔhfsH* pMR10:P_*Cu*_*-hfsH* in microfluidic channels with holdfast labeled with AF488-WGA (green). Exponential cultures were induced with 0 μM, 50 μM, or 250 μM CuSO_4_, mixed with AF488-WGA, injected into the microfluidic chambers, and allowed to bind for 30 min. Thereafter, the flow rate was adjusted to 1.4 ul/min. Images were collected every 20 s for 1 h. Blue arrows indicate shed holdfasts. Scale bar, 2 μm. See also Videos S5, S6, and S7.

To understand the role of HfsH in *H. baltica,* we investigated whether varying the level of its expression in *H. baltica* affects holdfast cohesive and adhesive properties. We used a copper-inducible promoter to tightly control the level of *hfsH* expression as shown on Figure 4A (Chepkwony et al., 2019). The ability to tightly regulate the expression levels of HfsH under the control of P_*Cu*_ was validated by Western blot analysis (Figure S3). We quantified the amount of biofilm formed after 4 h of *hfsH* induction with different concentrations of CuSO_4_ as an inducer of *hfsH* expression*.* Our results showed that the level of *hfsH* expression in *H. baltica* correlated logarithmically with the relative level of biofilm formed (Figure 4B). At the highest level of induction at 250 μM CuSO_4_, we observed full restoration of biofilm formation to WT levels (Figure 4B).

We next labelled holdfasts with AF488-WGA to analyze the effect of varying HfsH expression on *H. baltica* holdfast production*.* We induced the expression of HfsH for 2 h in *H. baltica* Δ*hfsH* P_*Cu*_*-hfsH* using 0 to 250 μM CuSO_4_ and visualized holdfasts of planktonic cells with AF488-WGA by fluorescence microscopy. Addition of CuSO_4_ to *H. baltica* WT and *H. baltica* Δ*hfsH* with empty vector controls had no effect on cell anchoring or holdfast surface adhesion (Figure 4C, upper and middle panels). In the *H. baltica* Δ*hfsH* mutant complemented with P_*Cu*_*-hfsH*, we observed a small area of AF488-WGA staining on cells and shed holdfasts in the medium at the lowest level of *hfsH* induction (10 μM CuSO_4_; Figure 4C, lower panels, blue arrow). As we increased the level of *hfsH* expression, we observed increasing levels of AF488-WGA labeling colocalized with cells (15% at 0 μM CuSO_4_ to 95% at 250 μM CuSO_4_ compared with WT, Figure 4C, lower panels). At intermediate levels of *hfsH* expression (50 μM CuSO_4_), we observed cells with labeled holdfasts but fewer rosettes than full induction at 250 μM CuSO_4_ (6% at 50 μM CuSO_4_ to 95% at 250 μM CuSO_4_, Figure 4C, lower panels). At the highest level of induction (250 μM CuSO_4_), we observed cells in rosettes and holdfast formation similar to WT (Figure 4C, lower panels, white arrow), suggesting that holdfast properties were fully restored at this level of HfsH expression.

To test if HfsH expression levels correlate with holdfast cohesiveness and adhesiveness, we performed time-lapse microscopy on *H. baltica* Δ*hfsH* complemented with pMR10:P_*Cu*_*-hfsH* grown in a microfluidic device. We induced the expression of HfsH for 2 h in liquid cultures, injected exponential-phase induced cells mixed with AF488-WGA into the microfluidic chamber, and turned off the flow for 30 min to allow for binding to the chamber surface. We then adjusted the flow rate to 1.4 μL/min and imaged the cells every 20 s for 30 min. *H. baltica* Δ*hfsH* pMR10:P_*Cu*_*-hfsH* grown without CuSO_4_ produced small holdfasts that adhered to the chamber, but were unable to anchor the cells to the surface once flow was turned back on (Figure 4D, upper panels and Video S5). Furthermore, holdfast material that was initially attached to the surface was subsequently washed away upon initiation of fluid flow, suggesting an adhesion defect (Figure 4D, upper panels, blue arrows). At 50 μM CuSO_4_, we observed a partial restoration of holdfast adhesiveness and cohesiveness as cells were able to stay attached to the surface for longer after reinitiation of the flow; however, holdfast adhesiveness was still impaired as shed holdfasts could be easily washed off the surface (Figure 4D, middle panels, blue arrows and Video S6). At 250 μM CuSO_4_*,* we observed full restoration of holdfast adherence, cell anchoring, and the formation of rosettes (Figure 4D, lower panels and Video S7). These results suggest that at lower levels of HfsH expression, holdfast binding properties are only partially restored, while at higher levels of expression, holdfast adhesiveness and cohesiveness are fully restored.

Video S5. HfsH expression correlates to the level of biofilm formation, related to Figure 4 Time-lapse videos of *H. baltica ΔhfsH* pMR10::P_*Cu*_*-hfsH* in microfluidic channels with holdfast labeled with AF488-WGA (green). Exponential cultures were induced with 0 μM CuSO_4_, mixed with AF488-WGA, injected into the microfluidic chambers, and allowed to bind for 30 min. Thereafter, the flow rate was adjusted to 1.4 ul/min. Images were collected every 20 sec for 1 h.

Video S6. HfsH expression correlates to the level of biofilm formation, related to Figure 4 Time-lapse videos of *H. baltica ΔhfsH* pMR10::P_*Cu*_*-hfsH* in microfluidic channels with holdfast labeled with AF488-WGA (green). Exponential cultures were induced with 50 μM CuSO_4_, mixed with AF488-WGA, injected into the microfluidic chambers, and allowed to bind for 30 min. Thereafter, the flow rate was adjusted to 1.4 ul/min. Images were collected every 20 sec for 1 h.

Video S7. HfsH expression correlates to the level of biofilm formation, related to Figure 4 Time-lapse videos of *H. baltica ΔhfsH* pMR10::P_*Cu*_*-hfsH* in microfluidic channels with holdfast labeled with AF488-WGA (green). Exponential cultures were induced with 250 μM CuSO_4_, mixed with AF488-WGA, injected into the microfluidic chambers, and allowed to bind for 30 min. Thereafter, the flow rate was adjusted to 1.4 ul/min. Images were collected every 20 sec for 1 h.

### Overexpression of HfsH increases biofilm formation in *C. crescentus* but not in *H. baltica*

**Figure 5:**
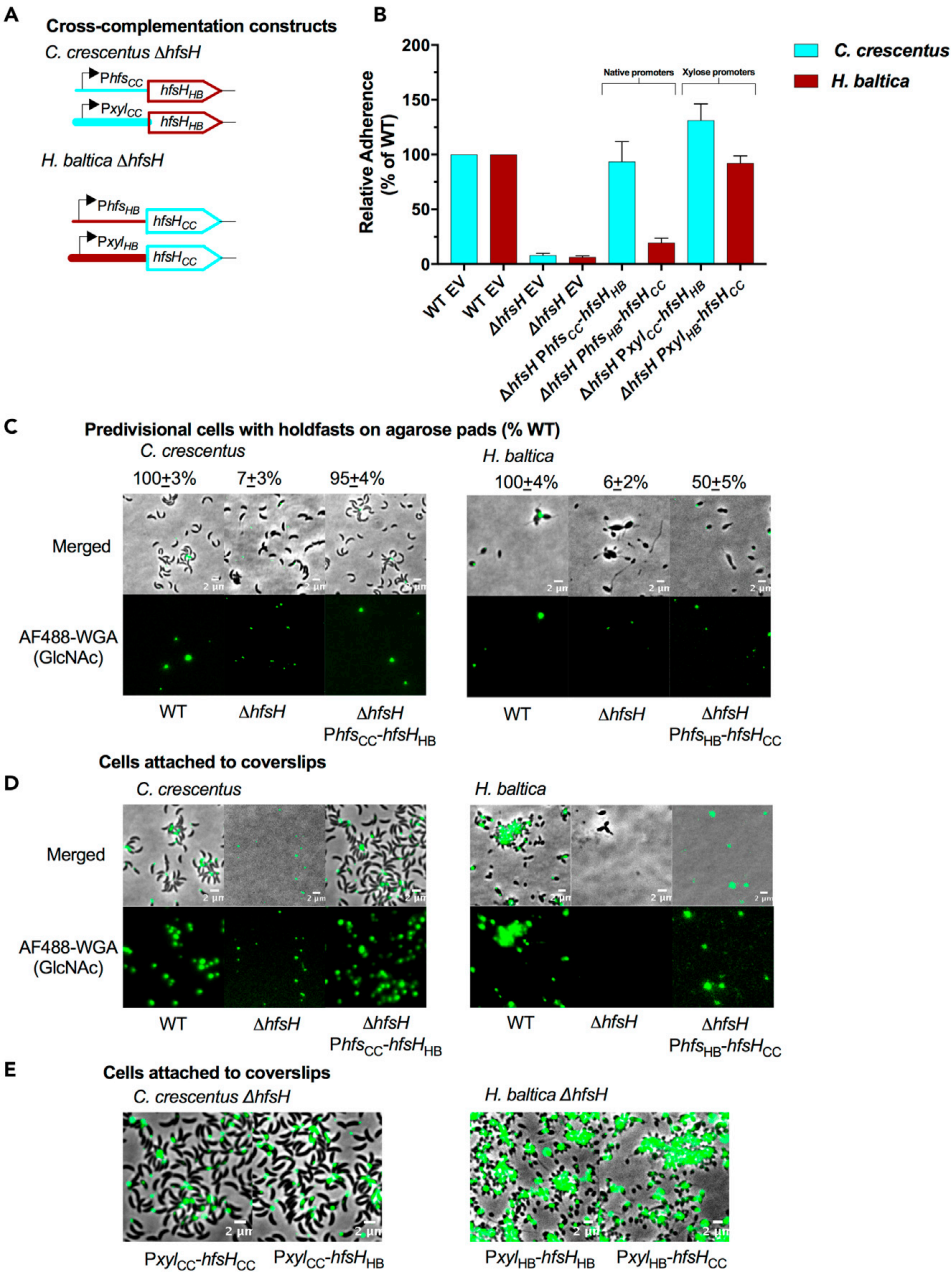
Overexpression of HfsH increases biofilm formation in *C. crescentus* but not *H. baltica* (A) Schematic representations of cross-complementation constructs of the *hfsH* gene from *C. crescentus* (*hfsH*_*CC*_) and *H. baltica* (*hfsH*_*HB*_) under native holdfast synthesis (P*hfs*) or xylose inducible (P*xyl*) promoters. Native promoters were fused to foreign *hfsH* genes (P*hfs*_*CC*_ and P*xyl*_*CC*_ from *C. crescentus*, or P*hfs*_*HB*_ and P*xyl*_*HB*_ from *H. baltica)* and assembled into the pMR10 plasmid. (B) Quantification of short-term adhesion (12 h) by the crystal violet assay. Data are expressed as a mean percent of WT crystal violet staining from three biological replicates with four technical replicates each. Error is expressed as the standard error of the mean. (C) Representative images showing merged phase and fluorescence channels of *C. crescentus* and *H. baltica* strains. Holdfasts are labeled with AF488-WGA (green). P*hfs*_CC_-*hfsH*_*HB*_, *C. crescentus ΔhfsH* cross*-*complemented with HfsH from *H. baltica* under the control of the *hfs* promoter; P*hfs*_HB_-*hfsH*_*CC*_, *H. baltica ΔhfsH cross-*complemented with HfsH from *C. crescentus* under the control of the *hfs* promoter. Exponential planktonic cultures were used to quantify the percentage of predivisional cells with holdfast. Scale bar, 2 μm. Data are expressed as the mean of three independent biological replicates with four technical replicates each. Error bars represent the standard error of the mean. (D) Images showing merged phase and fluorescence channels of *C. crescentus* and *H. baltica* strains bound to glass slides. Holdfast is labeled with AF488-WGA (green). Exponential cultures were incubated on the glass slides for 1 h, unbound cells were washed off, and AF488-WGA was added to label bound holdfast. Scale bar, 2 μm. (E) Merged phase and fluorescence channels of *H. baltica ΔhfsH* and *C. crescentus ΔhfsH* strains bound to glass slides. Holdfast is labeled with AF488-WGA (green). Exponential cultures were incubated on the glass slides for 1 h, unbound cells were washed off, and AF488-WGA was added to label bound holdfast. Strains carry native or cross-complemented HfsH under the control of the xylose inducible promoter for overexpression. Scale bar, 2 μm.

*C. crescentus* holdfast binding properties can be increased by overexpressing HfsH (Wan et al., 2013), implying that holdfast polysaccharides are partially deacetylated. *C. crescentus* Δ*hfsH* and *H. baltica* Δ*hfsH* showed important differences in their holdfast structure and binding properties (Figures 2C and 2D). Therefore, we hypothesized that there could be differences in the level of holdfast polysaccharide deacetylation among the Caulobacterales species. Unfortunately, because it is produced in such small quantities, it is not currently possible to directly determine the level of deacetylation in holdfasts. As an alternative, we examined the effect of varying the level of HfsH expression on holdfast adhesive properties. We first tested whether heterologous expression of HfsH in each species would alter holdfast properties. We made two types of cross-complementation constructs for each respective host species: (1) native levels of *hfsH* expression driven by the *hfsH* promoter (P*hfsH*_*CC*_ for *C. crescentus ΔhfsH* and P*hfsH*_*HB*_ for *H. baltica ΔhfsH*) and (2) *hfsH* overexpression driven by the inducible xylose promoter (P*xyl*_*CC*_ and P*xyl*_*HB*_; Figure 5A). We analyzed the level of HfsH expression by Western blot analysis and found that both HfsH_HB_ and HfsH_CC_ were equally expressed from these promoters (Figure S4).

To test whether the cross-complemented strains restored biofilm formation, we quantified biofilm formed after 12 h (Figure 5B). When we examined cross-complemented strains with HfsH under the control of the native promoter P*hfs*, we observed that P*hfs*_*CC*_*-hfsH*_*HB*_ restored biofilm formation to WT levels in *C. crescentus* Δ*hfsH* (Figure 5B). In contrast, P*hfs*_*HB*_*-hfsH*_*CC*_ restored biofilm formation to only 20% of WT levels in *H. baltica ΔhfsH* (Figure 5B). When *hfsH*_*HB*_ was overexpressed in *C. crescentus* from the P*xyl*_*CC*_ promoter (*C. crescentus ΔhfsH* P*xyl*_*CC*_*-hfsH*_*HB*_), biofilm formation was increased to 150% of WT levels (Figure 5B), similar to what has been observed with overexpression of HfsH_CC_ in *C. crescentus* (Wan et al., 2013). Overexpression of HfsH_CC_ using P*xyl*_*HB*_ in *H. baltica* Δ*hfsH* (*H. baltica ΔhfsH* P*hfs*_*HB*_*-hfsH*_*CC*_) restored biofilm to WT levels (Figure 5B). These results suggest that HfsH_HB_ may have higher levels of enzymatic activity than HfsH_CC_.

To analyze how cross-complementation of HfsH affects holdfast cohesion and anchoring, we labelled holdfasts from planktonic cultures with AF488-WGA. *C. crescentus ΔhfsH* P*hfs*_*CC*_-*hfsH*_*HB*_ holdfasts were labelled with AF488-WGA and formed rosettes similar to the WT (95% of WT, Figure 5C, left panels). As expected, *C. crescentus ΔhfsH* cross-complemented with P*hfs*_*CC*_-*hfsH*_*HB*_ produced holdfasts that bound to the coverslip and anchored the cells to the surface, similar to WT (Figure 5D, left panels). In *H. baltica ΔhfsH* P*hfs*_*HB*_*-hfsH*_*CC*_, we observed that approximately half of the stalked cells were labelled with AF488-WGA; however, this labelling was weaker than the WT (50% of WT, Figure 5C, right panels)*.* These results indicate that P*hfs*_*CC*_-*hfsH*_*HB*_ fully cross*-*complements *C. crescentus* Δ*hfsH*, while P*hfs*_*HB*_-*hfsH*_*CC*_ only partially cross-complements *H. baltica ΔhfsH.* These results are in agreement with our observations for biofilm formation for these strains (Figure 5B).

Because only half of *H. baltica* Δ*hfsH* P*hfs*_*HB*_-*hfsH*_*CC*_ cells had faint AF488-WGA labelling and surface-binding properties were not fully restored to WT levels (Figure 5C, right panels), we hypothesized that holdfasts produced by this mutant may have been shed into the medium. Therefore, we tested whether the holdfast produced by this strain can bind to a glass surface by incubating cells on a coverslip at room temperature for 1 h. After incubation, unattached cells were washed off and AF488-WGA was added to label any holdfast bound to the coverslip. We observed that holdfasts produced by *H. baltica ΔhfsH* P*hfs*_*HB*_-*hfsH*_*CC*_ were not able to anchor the cells to the glass surface, although these holdfasts were able to bind to the coverslip (Figure 5D, right panels). These results imply that expression of HfsH_CC_ from the *H. baltica hfsH* promoter was sufficient for restoration of holdfast surface binding by *H. baltica*, but insufficient to maintain interactions with the cell body. In addition, overexpression of either HfsH_CC_ or HfsH_HB_ in *C. crescentus* Δ*hfsH* and *H. baltica* Δ*hfsH* using P*xyl* restored holdfast binding properties to WT levels (Figure 5E). These results suggest either that HfsH_HB_ and HfsH_CC_ have different levels of enzymatic activity or that their ability to deacetylate *H. baltica* holdfast is different, which could be contributing to the observed differences in *C. crescentus* and *H. baltica* holdfast binding properties.

### Increased HfsH expression improves binding in high-ionic-strength environments

**Figure 6:**
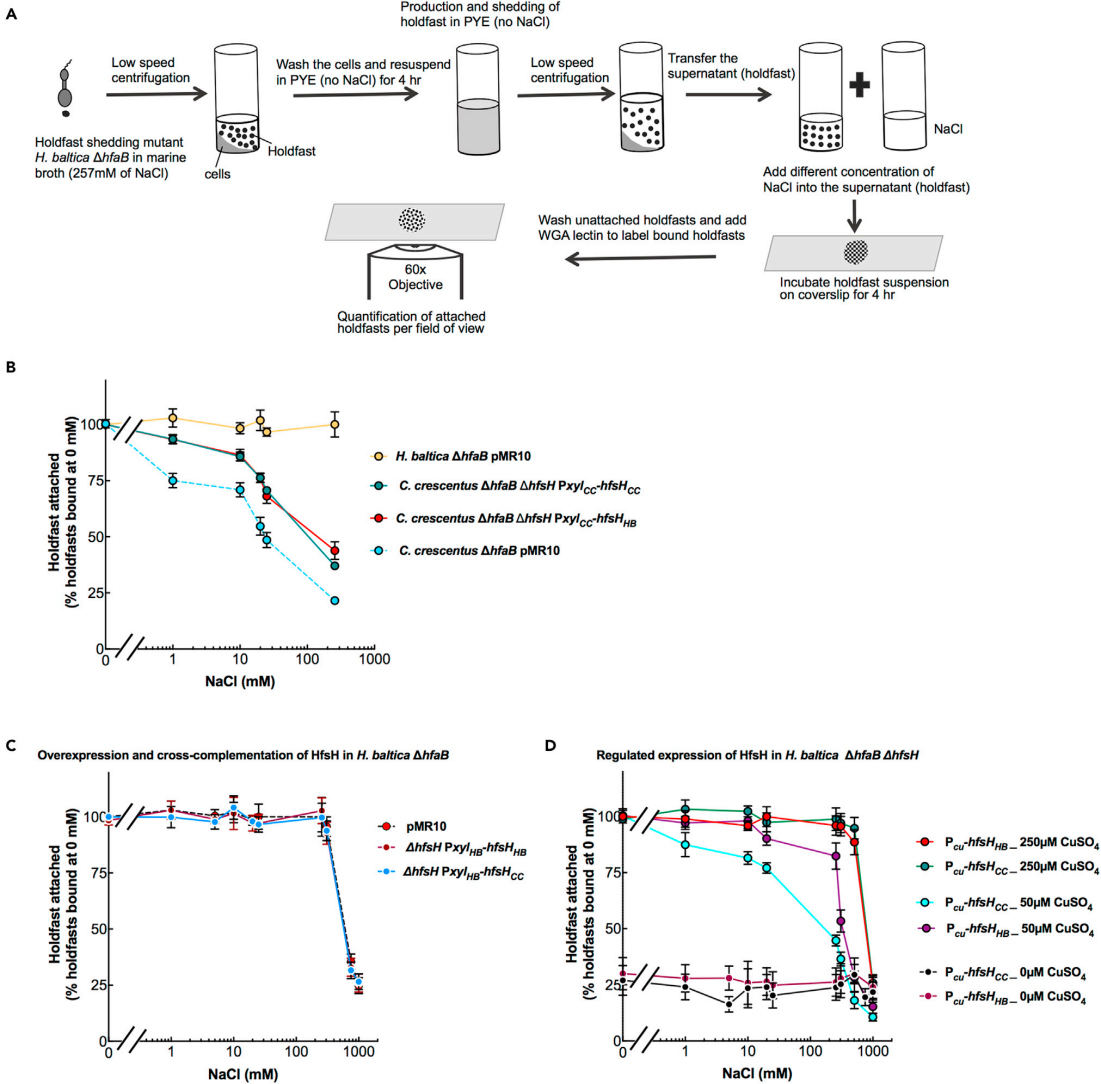
Increased holdfast deacetylation increases holdfast binding in high-ionic-strength environments (A) Schematic of the experimental setup. (B–D) Cells were grown exponentially for 2 h in PYE with 0.03% xylose (A-B), or 0 μM, 50 μM, or 250 μM CuSO_4_ (C) and shed holdfast were collected from the culture supernatant. The purified holdfasts were mixed with different concentration of NaCl and incubated on glass slides for 4 h. The percentage of holdfasts bound per field of view was quantified at different concentrations of NaCl. The number of holdfasts bound per field of view at 0 mM NaCl was standardized to 100%. Data are expressed as an average of five independent biological replicates with five technical replicates each. Error bars represent the standard error of the mean. (B) Purified holdfasts from *C. crescentus ΔhfaB* with pMR10 (empty vector, blue dashed line) as a control, *C. crescentus ΔhfaB ΔhfsH* complemented with HfsH from *C. crescentus* under the control of the xylose-inducible promoter (P*xyl*_CC_-*hfsH*_*CC*_, green), *C. crescentus ΔhfaB ΔhfsH* cross-complemented with HfsH from *H. baltica* under the control of the xylose-inducible promoter (P*xyl*_CC_-*hfsH*_*HB*_, red), and *H. baltica ΔhfaB* with pMR10 (empty vector, yellow). (C) Purified holdfasts from *H. baltica ΔhfaB* with pMR10 (empty vector, black dashed line) as a control, *H. baltica ΔhfaB ΔhfsH* complemented with HfsH_HB_ under the control of the xylose-inducible promoter (P*xyl*_HB_-*hfsH*_*HB*_, maroon), and *H. baltica ΔhfaB ΔhfsH* cross*-*complemented with HfsH_CC_ under the control of the xylose-inducible promoter (P*xyl*_HB_-*hfsH*_*CC*_, blue). (D) Purified holdfasts from *H. baltica ΔhfaB ΔhfsH* complemented with HfsH_HB_ under the control of the copper-inducible promoter (P_*Cu*_-*hfsH*_*HB*_) and *H. baltica ΔhfaB ΔhfsH* cross*-*complemented with HfsH_CC_ under the control of the copper-inducible promoter (P_*Cu*_-*hfsH*_*CC*_). P_*Cu*_-*hfsH*_*CC*_ was induced with 0 μM (black), 50 μM (blue), and 250 μM CuSO_4_ (green), and P_*Cu*_-*hfsH*_*HB*_ was induced with 0 μM (maroon), 50 μM (purple), and 250 μM CuSO_4_ (red).

To test whether HfsH plays an important role in holdfast binding at high ionic strength, we quantified binding of holdfasts purified from *C. crescentus* overexpressing HfsH_CC_ using the xylose promoter (P*xyl*_*CC*_*-hfsH*_*CC*_). To obtain cell-free holdfast samples and study holdfast without the contribution of the cell body, a holdfast-shedding mutation, Δ*hfaB*, was used as shown in Figure 6A (Hardy et al., 2010; Berne et al., 2010). Holdfasts from *C. crescentus* Δ*hfaB* Δ*hfsH* P*xyl*_*CC*_*-hfsH*_*CC*_ (Figure 6B, green line) tolerated higher ionic strengths than holdfasts purified from the control *C. crescentus* Δ*hfaB* strain (Figure 6B, blue dashed line). These results suggest that increased expression of HfsH in *C. crescentus* improves ionic strength tolerance. When we overexpressed HfsH_HB_ in *C. crescentus* Δ*hfaB* Δ*hfsH*, we observed an increase in ionic strength tolerance similar to overexpression of HfsH_CC_, but not to the level observed in *H. baltica* (Figure 6B, red, green and yellow lines). Our results suggest that holdfast polysaccharide deacetylation plays an important role in improving holdfast ionic strength tolerance, but it is not the only factor as increased HfsH_CC_ or HfsH_HB_ expression did not elevate *C. crescentus* holdfast binding to the level of *H. baltica*.

We next examined the effect of cross-complementation with HfsH_CC_ in *H. baltica.* We had observed that expression of HfsH_CC_ in *H. baltica ΔhfsH* from P*hfs*_*HB*_ failed to restore holdfast binding, but its overexpression using P*xyl* restored surface binding to the level observed in the WT (Figures 5A and 5B). Therefore, we tested how holdfasts purified from *H. baltica* Δ*hfaB* overexpressing HfsH_CC_ responded to ionic strength. *H. baltica* Δ*hfaB* Δ*hfsH* P*xyl*_*HB*_*-hfsH*_*CC*_ produced holdfasts that bound to glass slides to the same degree as holdfasts produced by control *H. baltica* Δ*hfaB* expressing regular levels of HfsH_HB_ (Figure 6C, black dashed line and blue line). We also quantified holdfast binding from *H. baltica ΔhfaB* overexpressing HfsH_HB_ and did not observe a further increase in ionic strength tolerance (Figure 6C, red line). These results suggest that *H. baltica* holdfasts either (1) have maximized binding at native levels of HfsH_HB_ expression or (2) have maximized holdfast deacetylation and further increases in HfsH_HB_ expression have no observable effects on holdfast binding.

We hypothesized that if increasing the level of HfsH expression increases *C. crescentus* holdfast binding at high ionic strength, then reducing the level of HfsH expression in *H. baltica* holdfast could reduce the ionic strength tolerance. To test this, we used the copper-inducible promoter P_*Cu*_ to control the expression of HfsH in *H. baltica* Δ*hfaB* Δ*hfsH.* We observed few holdfasts bound to the glass slide when HfsH_CC_ or HfsH_HB_ expression was not induced (Figure 6D, maroon and black lines). However, at the highest level of induction of HfsH_HB_ and HfsH_CC_ (250 μM CuSO_4_), we observed full restoration of ionic strength tolerance (Figure 6D, red and green lines). Next, we analyzed holdfast binding at an intermediate level of induction (50 μM CuSO_4_) because at this level of expression, we had observed 50% biofilm restoration and restoration of holdfast structure (Figures 4B and S5A). Using Western blot analysis, we compared the level of expression of HfsH_HB_ and HfsH_CC_ at 50 μM CuSO_4_ and observed that they were expressed at similar levels (Figure S5B). At intermediate levels of induction of HfsH_HB_, we observed a decrease in holdfast ionic strength tolerance compared with induction at 250 μM CuSO_4_ (Figure 6D, purple curve). We observed a further decrease in holdfast ionic strength tolerance when HfsH_CC_ was induced with 50 μM CuSO_4_ compared with HfsH_HB_ (Figure 6D, blue and purple curves). The effect of reducing HfsH expression was larger at high ionic strength than at low ionic strength, suggesting that holdfast polysaccharide deacetylation may play an important role in promoting holdfast binding at high ionic strength (Figure 6D). These results suggest that HfsH_HB_ likely deacetylates *H. baltica* holdfast more efficiently than HfsH_CC_ and that marine Caulobacterales have optimized HfsH to augment holdfast binding at high ionic strength.

### HfsH is required for retention of holdfast thiols and galactose monosaccharides

In addition to polysaccharides, the *H. baltica* holdfast contains free thiol groups, suggesting that it contains proteins (Chepkwony et al., 2019). Holdfast thiols require the presence of holdfast polysaccharides for cell association, as deletion of the glycosyltransferases essential for holdfast polysaccharide synthesis leads to loss of both holdfast polysaccharides and thiols (Chepkwony et al., 2019). In addition to GlcNAc, *H. baltica* holdfasts contain galactose monosaccharides (Chepkwony et al., 2019). To gain insights into how HfsH modifies holdfast properties, we analyzed its impact on these holdfast components.

**Figure 7:**
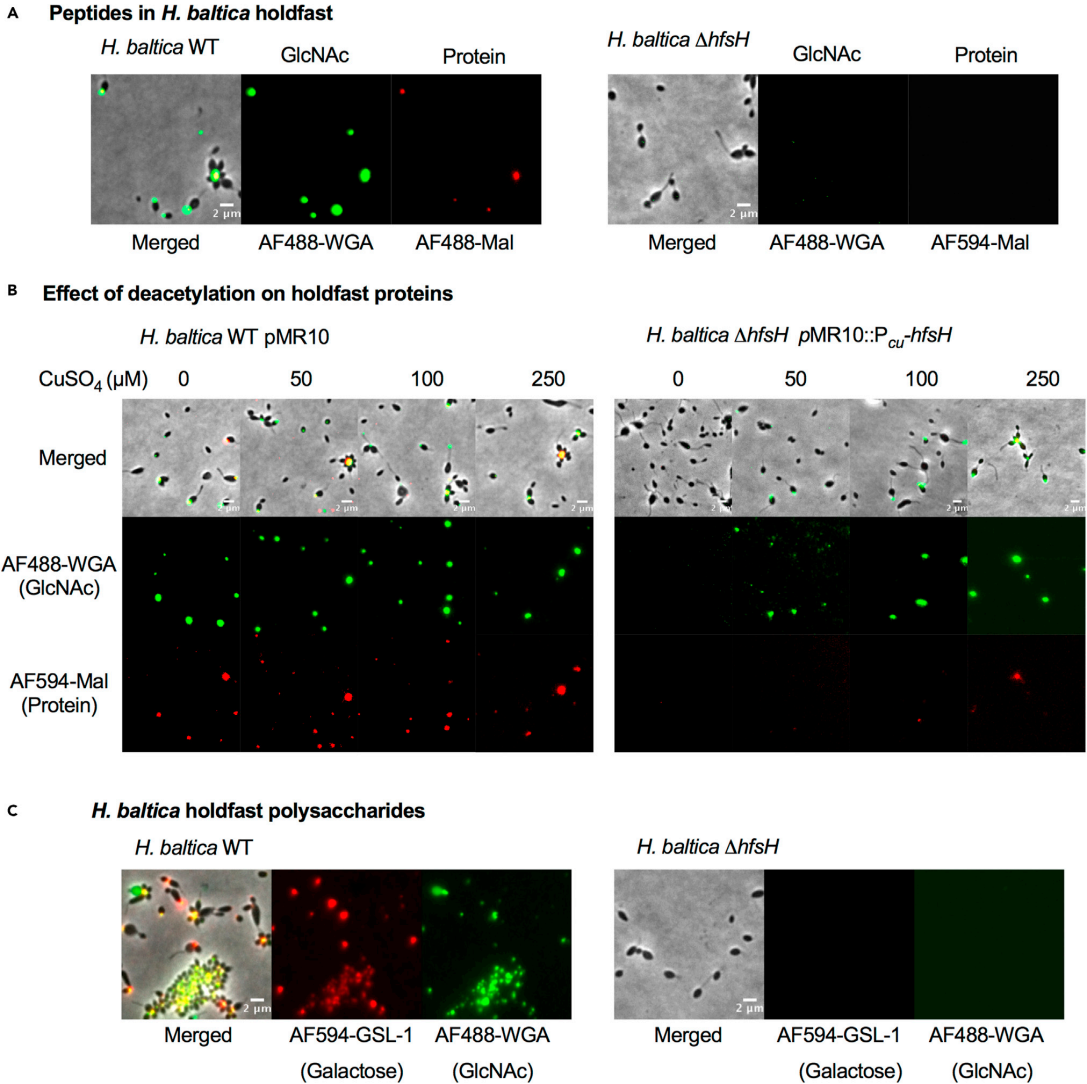
HfsH expression is required for interaction of holdfast thiols and galactose monosaccharides with cells (A) Representative images showing merged phase and fluorescence channels of *H. baltica* and *H. baltica ΔhfsH* holdfasts colabeled with AF488-WGA (green, GlcNAc) to label polysaccharides and AF594-mal (red) to label free thiols. Scale bar, 2 μm. (B) Representative images of *H. baltica* WT *and H. baltica ΔhfsH* complemented with pMR10:P_*Cu*_-*hfsH*. Holdfasts were colabeled with AF488-WGA (green) and AF594-Mal (red). Exponential cultures were induced for 2 h with concentrations of CuSO_4_ ranging from 0–250 μM. Scale bar, 2 μm. (C) Representative images showing merged phase and fluorescence channels of *H. baltica* and *H. baltica ΔhfsH* holdfasts colabeled with AF488-WGA (green) and AF594-GSL-1 (red) to label GlcNAc and galactose in holdfast, respectively. Scale bar, 2 μm.

To test whether holdfast thiols are present in the deacetylase mutant *H. baltica* Δ*hfsH*, we colabeled exponential-phase cells with both AF488-WGA (green, GlcNAc) and AF594 conjugated to maleimide (AF594-Mal), which reacts with free thiol molecules (red). As expected, the WT cells were labeled with both AF488-WGA and AF594-Mal (Figure 7A, left panels), indicating the presence of both holdfast polysaccharides and thiols. In contrast, the deacetylase mutant *H. baltica* Δ*hfsH* was not labelled with either AF488-WGA or AF594-Mal (Figure 7A, right panels). We then varied the level of HfsH expression using *H. baltica* Δ*hfsH* P_*Cu*_-*hfsH*. Addition of CuSO_4_ to exponentially growing *H. baltica* WT cells with empty vector had no effect on labeling of holdfast polysaccharides (Figure 7B, left panels). At the lowest level of induction (50 μM CuSO_4_), *H. baltica* Δ*hfsH* P_*Cu*_*-hfsH* holdfasts were labeled only by AF488-WGA while AF594-Mal failed to label holdfasts (Figure 7B, right panels). We observed restored AF594-Mal labeling at the highest level of HfsH induction (250 μM CuSO_4_; Figure 7B, right panels). These results indicate that HfsH expression is required for thiol-containing molecules to associate with GlcNAc polysaccharides in the holdfast.

To test the effect of *hfsH* deletion on retention of the galactose component of holdfast, we colabeled the cells with AF488-WGA (specific to GlcNAc, green) and AF594-conjugated *Griffonia simplicifolia* lectin 1 (AF594-GSL-1, specific to galactose, red). *H. baltica* WT holdfast was labelled with both AF488-WGA and AF594-GSL-1, but *H. baltica ΔhfsH* was not labeled with either AF594-GSL-1 or AF488-WGA (Figure 7C). These results suggest that HfsH is also crucial for the retention of holdfast galactose components within the holdfast of *H. baltica*.

## Discussion

Bacterial adhesion is influenced by many variables, including adhesin composition, surface properties, and environmental factors such as pH, temperature, fluid shear, and ionic strength (Abu-Lail and Camesano, 2003a, 2003b; Berne et al., 2013, 2018; Donlan, 2002; Dunne, 2002). *C. crescentus,* a freshwater Caulobacterales, produces holdfasts that are sensitive to ionic strength, while holdfasts from the marine *H. baltica* tolerate a higher ionic strength (Berne et al., 2013; Chepkwony et al., 2019). In this study, we examined the influence of specific enzymatic modifications on the differing holdfast properties of these species. Specifically, we described the contributions of the polysaccharide deacetylase HfsH to holdfast binding at high ionic strength by comparing holdfasts from the freshwater *C. crescentus* and the marine *H. baltica.* The degree of deacetylation modifies holdfast polysaccharide physicochemical properties by introducing a partially positive charge on the resultant amine group, which is important for holdfast cohesive and adhesive properties (Wan et al., 2013).

Our results showed that HfsH is important for biofilm formation and holdfast binding properties in *H. baltica*. We found that the *H. baltica* Δ*hfsH* mutant does not form rosettes or biofilms and produces holdfasts that are impaired in surface binding and have a thread-like appearance, in contrast to the *C. crescentus* Δ*hfsH* mutant, which sheds small holdfasts that are capable of binding to glass surfaces. These observations suggest that there are differences in the role for holdfast deacetylation in *H. baltica versus C. crescentus.* These results also indicate that HfsH plays an important role in maintaining holdfast cohesive and adhesive properties in both species. It has been shown that polymers such as xylan and lignocellulose interact with surfaces using hydrogen bonds generated by deacetylation and the degree of deacetylation affects their interactions with polar surfaces (Pawar et al., 2013). The degree of deacetylation of other polysaccharides such as chitin/chitosan, a polymer of GlcNAc, is important in altering their physicochemical properties. For example, deacetylation of chitin to generate chitosan increases its pK_a_ from 6.46 to 7.32 (Sorlier et al., 2001). This pK_a_ change is due to an increase in free primary amines that are exposed by deacetylation. We believe that the polysaccharide deacetylase HfsH performs a similar function in modifying the holdfast polysaccharide.

Overexpression of HfsH_CC_ in *C. crescentus* increases holdfast adhesion without an increase in the size of holdfast (Wan et al., 2013). This suggests that *C. crescentus* holdfast polysaccharides are partially deacetylated and overexpression of HfsH_CC_ further increases the degree of deacetylation, in turn enhancing adhesion. In *H. baltica,* overexpression of HfsH_HB_ or HfsH_CC_ did not increase surface adhesion compared with WT. One interpretation of these results is that *H. baltica* holdfast polysaccharides are fully deacetylated by native levels of HfsH expression, and thus, overexpression of HfsH has no additional effect. Alternatively, *H. baltica* holdfast binding is already maximized for out test conditions and thus an increase in deacetylation has no further positive effect. We hypothesized that if *H. baltica* has maximized its holdfast polysaccharide deacetylation, expression of HfsH below native levels would lead to a reduction in holdfast ionic strength tolerance. We showed that the level of HfsH expression correlates with holdfast binding in *H. baltica,* suggesting that *H. baltica* is exploiting deacetylation to optimize its binding in high-ionic-strength environments. Our results showed that HfsH_HB_ performs this function better than HfsH_CC,_ suggesting that there could be differences in HfsH_HB_ and HfsH_CC_ enzymatic activities. *H. baltica* produces larger holdfasts than *C. crescentus,* and they contain additional sugars such as galactose (Chepkwony et al., 2019); therefore, an alternative hypothesis is that *C. crescentus* HfsH_CC_ might be less efficient at deacetylating *H. baltica* holdfast owing to structural and compositional differences.

Interestingly, increasing expression of HfsH in *C. crescentus* leads to increased binding at high ionic strength. Cross-complementing *C. crescentus* Δ*hfsH* with overexpressed HfsH_HB_ produced holdfasts that had similar levels of increased ionic strength tolerance as those produced by overexpressed HfsH_CC_ as compared with WT, suggesting that HfsH_HB_ is capable of deacetylating *C. crescentus* holdfast polysaccharides. However, increased expression of HfsH_HB_ did not increase *C. crescentus* holdfast binding at high ionic strength to the level of *H. baltica,* implying that other factors also contribute to ionic strength tolerance. When we reduced the expression of HfsH_HB_ in *H. baltica,* we observed a decrease in ionic strength tolerance. A further decrease was observed when HfsH_CC_ was expressed at the same intermediate level in *H. baltica* compared with HfsH_HB_. These results suggest that for *H. baltica* holdfast to overcome high ionic strength, there is a minimum level of deacetylation of holdfast polysaccharides that must be attained.

How holdfast interacts with surfaces remains unclear, but an electrostatic mechanism has been suggested (Berne et al., 2013; Chepkwony et al., 2019). *C. crescentus* holdfast binding is affected by pH and NaCl (Berne et al., 2013). The mechanism by which NaCl disrupts electrostatic interactions between holdfast components and glass surfaces is unclear. High ionic strength has been shown to reduce the radius of the electrostatic force on a surface, which would lower the likelihood that holdfast polysaccharides are able to interact with the surface (Chen and Walker, 2007). It is also known that increasing ionic strength has no effect on holdfast that are already attached to a surface (Chepkwony et al., 2019), suggesting that high ionic strength only impairs the initial interactions between the holdfast and the surface before a permanent bond is established. In *Pseudomonas putida*, it has been shown that high ionic strength alters the conformation of extracellular biopolymers (Abu-Lail and Camesano, 2003a, 2003b; Shephard et al., 2010). The polymer brush layer remains extended at low ionic strength, but upon an increase in ionic strength, the brush layer becomes compacted, leading to an increase in the charge-to-mass ratio (Abu-Lail and Camesano, 2003a, 2003b; Shephard et al., 2010). This increase in charge-to-mass ratio ensures that the polysaccharides retain their electrostatic properties. This phenomenon could explain the need for a higher level of deacetylation of holdfasts from marine species than for those of freshwater species, as deacetylation increases the proportion of charges on holdfast polysaccharides, as required for surface interactions at high ionic strength.

We conclude that the degree of holdfast polysaccharide deacetylation is important in holdfast binding at high ionic strengths and that the marine Caulobacterales have optimized deacetylation to overcome holdfast binding challenges in these environments. Generally, it seems like the degree of holdfast deacetylation and the degree of 3,4-dihydroxyphenyl-L-alanine (DOPA) incorporation into the Mfps are equivalent strategies to adapt to increased ionic strength. We showed that the *H. baltica* Δ*hfsH* mutant lacks both galactose and thiol molecules, suggesting that these constituents require deacetylated holdfast to associate with the cell. Therefore, deacetylated GlcNAc, sugars other than GlcNAc, and/or putative thiol-containing proteins might play a role in improving holdfast binding at high ionic strength. Validation of the presence of putative holdfast-associated proteins and their identification in *C. crescentus* and *H. baltica* will enable a better understanding of their role in holdfast binding in these environments.

### Limitations of the study

A limitation of this study is that we were not able to purify *H. baltica* HfsH to test its deacetylase activity. Instead, we obtained genetic data supporting deacetylase activity of HfsH. We showed that in both strains, the D48A/D43A active site mutation phenocopied the *ΔhfsH* mutant. Cross-complementation of *C. crescentus ΔhfsH* with *hfsH*_*HB*_ restored holdfast binding properties. However, cross-complementation of *C. crescentus ΔhfsH* with the active site mutant *hfsH*_*HB*_^*D43A*^ failed to restore holdfast binding properties. Because we had showed in previous work that *C. crescentus* HfsH has deacetylase activity that is abolished by the equivalent mutation (Wan et al., 2013), these data provide strong genetic support for *H. baltica* HfsH deacetylase activity. In addition, we were unable to measure the level of holdfast polysaccharide deacetylation owing to the small amounts of holdfast produced by cells and the difficulty of working with this highly adherent material.

## STAR★Methods

### Key resources table

**Table:** 

REAGENT or RESOURCE	SOURCE	IDENTIFIER
**Antibodies**		

α-FLAG (M2)	Millipore Sigma	Cat# A8592: RRID:AB_439702
α-McpA	Brun Lab	N/A
Goat Anti-Mouse IgG HRP conjugate	Millipore Sigma	Cat# AP130P, RRID:AB_91266

**Bacterial strains**		

*Caulobacter crescentus* strains, see Table S1	This study, Poindexter, 1964, Sprecher et al.2017, Toh et al., 2008, Chepkwony et al., 2019 and Hardy et al., 2010	N/A
*Hirschia baltica* strains, see Table S1	This study, Chepkwony et al., 2019 and Schlesner et al., 1990	N/A

**Chemicals, peptides, and recombinant proteins**		

Alexa Flour conjugated Maleimide C_5_ (AF488-mal)	ThermoFisher Scientific	Cat# A10254
Alexa Flour conjugated wheat germ agglutinin (AF594WGA and AF488WGA)	Molecular Probes	Cat# W11261, W11262
Alexa Flour conjugated conjugated *Griffonia simplicifolia* l (AF594GSL1)	Molecular Probes	Cat# L21416
EcoRV-HF endonuclease	New England Biolabs	Cat# RS3195S
NEBuilder® HiFi DNA Assembly master mix	New England Biolabs	Cat# E5520S
SuperSignal West Dura Substrate	Thermo Scientific	Cat# 34075
Difco^TM^ Marine Broth/Agar	BD	reference 2216/
Peptone Yeast Extract (PYE)	BD, Poindexter, 1964	Ref 211677, 212750,214010
Lysogeny broth (LB)	BD, Bertani G., 1951	Ref 211705, 214010, 214010
Kanamycin Sulfate	VWR	CAS# 25389-94-0

**Experimental models: organisms/strains**		

*E. coli* α-select competent cells	Bioline	Cat# Bio-85025
*E. coli* BL21(DE3)	New England Biolabs	Cat# 69450

**Oligonucleotides**		

Primers for gene deletion, complementations and overexpression of *hfsH*, see Table S2	This paper	N/A

**Recombinant DNA**		

pET28a(+)	Novagen	Cat# 69865
pET28a *hfsH*_*CC*_	This study	N/A
pET28a *hfsH*_*HB*_	This study	N/A
pMR10	R. Roberts and C. Mohr	N/A
pMR10 with gene constructs, see Table S1	This Study	N/A
pNPTS139	M.R..K Alley	N/A
pNPTS139 with gene constructs, see Table S1	This study	N/A

**Software and algorithms**		

ImageJ	Schneider et al., 2012	https://imagej.nih.gov/ij/
MicrobeJ	Ducret et al., 2016	https://www.nature.com/articles/nmicrobiol201677
GraphPad Prism	GraphPad Software, LLC	https://www.Graphpad.com

### Resource availability

#### Lead contact

Further information and requests for resources and reagents should be directed to and will be fulfilled by the lead contact, Yves V. Brun, yves.brun@umontreal.ca.

#### Materials availability

This study did not generate new unique reagents or genetically modified organisms.

### Experimental model and subject details

#### Bacterial strains and growth conditions

The bacterial strains used in this study are listed in Table S1. *H. baltica* strains were grown in marine medium (Difco^TM^ Marine Broth/Agar reference 2216) except when studying the effect of ionic strength on holdfast binding, where they were grown in Peptone Yeast Extract (PYE) medium (Poindexter, 1964) supplemented with 0 or 1.5% NaCl. *C. crescentus* was grown in PYE medium. Both *H. baltica* and *C. crescentus* strains were grown at 30°C. When appropriate, kanamycin was added at 5 μg/ml to liquid medium and 20 μg/ml in agar plates. *H. baltica* strains with the copper inducible promoter were grown in marine broth supplemented with 0–250 μM of CuSO_4_. *H. baltica* strains with the xylose promoter were grown in marine broth supplemented with 0.03% xylose, while *C. crescentus* strains with the xylose promoter were grown in PYE broth supplemented with 0.03% xylose. *E. coli* strains were grown in lysogeny broth (LB) at 37°C supplemented with 30 μg/ml of kanamycin in liquid medium or 25 μg/ml in agar plates, as appropriate.

#### Strain construction

All the plasmids and primers used in this study are listed in Tables S1 and S2, respectively. In-frame deletion mutants were obtained by double homologous recombination as previously described (Ried and Collmer, 1987) using suicide plasmids transformed into the *H. baltica* host strains by electroporation (Ely, 1991) followed by sacB sucrose selection. Briefly, genomic DNA was used as the template to PCR-amplify 500 bp fragments immediately upstream and downstream of the gene to be deleted. The primers used for amplification were designed with 25 bp overlapping segments for isothermal assembly (Gibson et al., 2009) using the New England Biolabs NEBuilder® HiFi tools for ligation into plasmid pNPTS139, which was digested using EcoRV-HF endonuclease from New England Biolabs. pNPTS139-based constructs were transformed into *α-*select *E. coli* for screening and sequence confirmation before introduction into the host *C. crescentus* or *H. baltica* strains by electroporation. Introduction of the desired mutation onto the *C. crescentus* or *H. baltica* genome was verified by sequencing.

For gene complementation, the pMR10 plasmid was cut with EcoRV-HF and 500 bp upstream of the gene of interest containing the promoter, as well as the gene itself, were designed using New England Biolabs NEBuilder® HiFi tools and fragments were amplified and ligated into plasmid pMR10 as described above. The pMR10-based constructs were transformed into *α-*select *E. coli* for screening and sequence confirmation before introduction into the host *C. crescentus* or *H. baltica* strains by electroporation.

### Method details

#### Holdfast labeling using fluorescent lectins

Holdfast labeling with AF488 conjugated lectins (Molecular Probes) was performed as previously described (Chepkwony et al., 2019) with the following modifications. Overnight cultures were diluted in fresh medium to an OD_600_ of 0.2 and incubated for 4 h to an OD_600_ of 0.6–0.8. AF488 conjugated lectins were added to 100 μl of the exponential culture to a final concentration of 0.5 μg/ml and incubated at room temperature for 5 min. 5 μl of the labeled culture was spotted onto a glass cover slide, overlaid with a 1.5% (w/v) agarose (Sigma-Aldrich) pad in water, and visualized by epifluorescence microscopy. Imaging was performed using an inverted Nikon Ti-E microscope with a Plan Apo 60X objective, a GFP/DsRed filter cube, an Andor iXon3 DU885 EM CCD camera, and Nikon NIS Elements imaging software with 200 ms exposure time. Images were processed in ImageJ (Schneider et al., 2012).

#### Short-term adherence and biofilm assays

This assay was performed as previously described (Chepkwony et al., 2019) with the following modifications. For short-term binding assays, exponential cultures (OD_600_ of 0.6–0.8) were diluted to an OD_600_ of 0.4 in fresh marine broth, added to 24-well plates (1 ml per well), and incubated with shaking (100 rpm) at room temperature for 4 h. For biofilm assays, overnight cultures were diluted to an OD_600_ of 0.1, added to a 24-well plate (1 ml per well), and incubated at room temperature for 12 hours with shaking (100 rpm). In both set-ups, OD_600_ was measured before the wells were rinsed with distilled H_2_O to remove non-adherent bacteria, stained using 0.1% crystal violet (CV), and rinsed again with dH_2_O to remove excess CV. The CV was dissolved with 10% (v/v) acetic acid and quantified by measuring the absorbance at 600 nm (A_600_). Biofilm formation was normalized to A_600_ / OD_600_ and expressed as a percentage of WT.

#### HfsH expression using a copper inducible promoter

Strains bearing copper inducible plasmids were inoculated from freshly grown colonies into 5 ml marine broth containing 5 μg/ml kanamycin and incubated with shaking (200 rpm) at 30°C overnight. Overnight cultures were diluted in fresh marine broth to OD_600_ of 0.1 and incubated until an OD_600_ of 0.4 was reached, where copper sulfate dissolved in marine broth was added to a final concentration of 0–250 μM. For holdfast labeling, AF488 conjugated lectins were added to 100 μl of exponential culture to a final concentration of 0.5 μg/ml and incubated at room temperature for 5 min. 5 μl of the labeled culture was spotted on glass cover slide, overlaid with a 1.5 % (w/v) agarose (Sigma-Aldrich) pad in water, and visualized by epifluorescence microscopy. Imaging was performed using an inverted Nikon Ti-E microscope with a Plan Apo 60X objective, a GFP/DsRed filter cube, an Andor iXon3 DU885 EM CCD camera and Nikon NIS Elements imaging software with 200 ms exposure time. Images were processed in ImageJ (Schneider et al., 2012).

For short term binding and biofilm assays, the induced cultures and controls (OD_600_ = 0.4) were incubated with shaking (100 rpm) at room temperature for 4–12 h. Then, OD_600_ was measured before the wells were rinsed with distilled H_2_O to remove non-adherent bacteria, stained using 0.1% crystal violet (CV), and rinsed again with dH_2_O to remove excess CV. The CV was dissolved with 10% (v/v) acetic acid and quantified by measuring the absorbance at 600 nm (A_600_) using Bio-Rad microplate reader (Bio-Rad Laboratories, Inc). Biofilm formation was normalized to A_600_/OD_600_ and expressed as a percentage of WT.

#### Visualization of holdfasts attached on a glass surface

Visualization of holdfast binding to glass surfaces was performed as described previously (Chepkwony et al., 2019) with the following modifications. *H. baltica and C. crescentus* strains grown to exponential phase (OD_600_ = 0.4–0.6) were incubated on washed glass coverslips at room temperature in a saturated humidity chamber for 4–8 h. After incubation, the slides were rinsed with dH_2_O to remove unbound cells, holdfasts were labeled using 50 μl of fluorescent AF488/594 conjugated lectins at a concentration of 0.5 μg/ml, and cover slides were incubated at room temperature for 5 min. Then, excess lectin was washed off and the cover slide was topped with a glass coverslip. Holdfasts were imaged by epifluorescence microscopy using an inverted Nikon Ti-E microscope with a Plan Apo 60X objective, a GFP/DsRed filter cube, an Andor iXon3 DU885 EM CCD camera and Nikon NIS Elements imaging software with 200 ms exposure time. Images were processed in ImageJ (Schneider et al., 2012).

#### Holdfast synthesis by time-lapse microscopy on soft agarose pads

*H. baltica* holdfast synthesis was observed in live cells on agarose pads by time-lapse microscopy as described previously (Chepkwony et al., 2019) with some modifications. A 1 μl aliquot of exponential-phase cells (OD_600_ of 0.4–0.8) induced with 0–250 μM CuSO_4_ was placed on top of a 0.8% agarose pad in marine broth with 0.5 μg/ml of AF488-WGA. The pad was overlaid with a coverslip and sealed with VALAP (Vaseline, lanolin and paraffin wax). Time-lapse microscopy images were taken every 5 min for 12 h using an inverted Nikon Ti-E microscope and a Plan Apo 60X objective, a GFP/DsRed filter cube, and an Andor iXon3 DU885 EM CCD camera. Time-lapse movies were processed using ImageJ (Schneider et al., 2012).

#### Holdfast synthesis in a microfluidic device by time-lapse microscopy

This experiment was performed as previously described (Chepkwony et al., 2019) with the following modifications. Cell cultures were grown to mid-exponential phase (OD_600_ = 0.4–0.6) and induced with 0–250 μM CuSO_4_. Then, 200 μl of culture was diluted into 800 μl of fresh marine broth with 0–250 μM CuSO_4_ in the presence of 0.5 μg/ml AF488-WGA for holdfast labeling. One ml of the cell culture was then flushed into a microfluidic device containing a 10 μm high linear chamber fabricated in PDMS (Polydimethylsiloxane) as described previously (Hoffman et al., 2015). After injection of the cells into the microfluidic chamber, the flow rate was adjusted so that attachment could be observed under static conditions or low flow rate of 1.4 μl/min.

Time-lapse microscopy was performed using an inverted Nikon Ti-E microscope and a Plan Apo 60X objective, a GFP/DsRed filter cube, an Andor iXon3 DU885 EM CCD camera, and Nikon NIS Elements imaging software. Time-lapse videos were collected over a period of 5.5 h at 20-second intervals. Cell attachment was detected at the glass-liquid interface within the microfluidic chamber using phase contrast microscopy, while holdfast synthesis was detected using fluorescence microscopy. Time-lapse movies were processed using ImageJ (Schneider et al., 2012).

#### Holdfast labeling using fluorescently labeled maleimide and lectin

Alexa Flour conjugated Maleimide C_5_ (AF488-mal, ThermoFisher Scientific) and AF594-WGA (Molecular Probes) were both added to 100 μl of exponential culture to a final concentration of 0.5 μg/ml and incubated at room temperature for 5 min. 5 μl of the labeled culture was spotted onto a glass cover slide, overlaid with a 1.5% (w/v) agarose (Sigma-Aldrich) pad in water, and visualized by epifluorescence microscopy. Imaging was performed using an inverted Nikon Ti-E microscope with a Plan Apo 60X objective, a GFP/DsRed filter cube, an Andor iXon3 DU885 EM CCD camera, and Nikon NIS Elements imaging software with 200 ms exposure time. Images were processed in ImageJ (Schneider et al., 2012).

#### Effect of ionic strength on holdfast binding

Visualization of attachment of purified holdfasts to surfaces at different ionic strengths was performed as described previously (Chepkwony et al., 2019) with the following modifications. Briefly, exponential cultures of strains carrying *hfsH* under the control of the copper inducible promoter (P_*Cu*_), xylose inducible promoters (P*xyl*), or controls (P*hfs*) were grown to late exponential phase (OD_600_ = 0.6–0.8) in PYE with 1.5% (w/v) NaCl for *H. baltica* strains, or PYE with no NaCl for *C. crescentus* strains with 0–250 μM CuSO_4_ or 0.03% xylose. The cells were collected by centrifugation for 30 min at 4,000 x *g* and resuspended in PYE with 0–250 μM CuSO_4_ or 0.03% xylose and incubated for 2 h at 30°C. Then, the cells were again collected by centrifugation as above and 100 μl of the resultant supernatant, containing holdfasts shed by the cells, were mixed with 100 μl of NaCl in PYE to a final concentration of 0–1000 mM of NaCl. 50 μl of the mixture was incubated on washed glass coverslips at room temperature in a saturated humidity chamber for 4–12 h. After incubation, the slides were rinsed with dH_2_O to remove unbound material and holdfast were visualized with AF conjugated lectins (Molecular Probes). Imaging was performed using an inverted Nikon Ti-E microscope with a Plan Apo 60X objective, a GFP/DsRed filter cube, an Andor iXon3 DU885 EM CCD camera, and Nikon NIS Elements imaging software with 200 ms exposure time. Images were processed in ImageJ (Schneider et al., 2012). The number of holdfasts bound per field of view was quantified using MicrobeJ (Ducret et al., 2016).

#### Western blot analysis

Cell lysates were prepared from exponentially growing cultures (OD_600_ = 0.6–0.8) as previously described (Wan et al., 2013) with the following modifications. The equivalent of 1.0 ml of culture at an OD_600_ of 0.6–0.8 was centrifuged at 16,000 × *g* for 5 min at 4°C. The supernatant was removed, and cell pellets were resuspended in 50 μl of 10mM Tris pH 8.0, followed by the addition of 50 μl of 2x SDS sample buffer. Samples were boiled for 5 min at 100°C before being run on a 12% (w/v) polyacrylamide gel and transferred to a nitrocellulose membrane. Membranes were blocked for 30 min in 5% (w/v) non-fat dry milk in TBST (20 mM Tris, pH 8, 0.05% (w/v) Tween 20), and incubated at 4°C overnight with primary antibodies. Anti-FLAG tag and McpA antibodies were used at a concentration of 1:10,000. Then, a 1:10,000 dilution of secondary antibody, HRP-conjugated goat anti-rabbit immunoglobulin, was incubated with the membranes at room temperature for 2 h. Membranes were developed with SuperSignal West Dura Substrate (Thermo Scientific, Rockford, IL).

### Quantification and statistical analysis

#### Biofilm quantification

The raw data from microplate reader (Bio-Rad Laboratories Inc.,) were used to calculate the percentage of cells bound to the wells after incubation using GraphPad Prism 8 (GraphPad Software LLC, San Diego, CA, USA, 2021). The WT values were normalized to 100%. The error on the bar graph is expressed as the standard error of the three independent biological replicates with four technical replicates each. Nonlinear regression was used to curve fit data for HfsH expression *vs* biofilm formation (Figure 4C). Unpaired t test (Two-tailed) was used to calculate statistical differences. Data analysis for each graph is found on the figure legend.

#### Holdfast and cell attachment to surfaces

ImageJ was used to count the number of holdfasts and cells bound to the glass slides using MicrobeJ plug-in. For each experiment, a total of 10 images were used and total 1–3000 cells and 100–1000 holdfasts were quantified on each of the two independent biological replicates. MicrobeJ was used to calculate mean and standard error of the three biological replicates with five technical replicates for Figures 2 and 4. Analyzed data were imported to GraphPad Prism 8 to generate a logarithmic graph for Figure 6 (error bars represent standard error of mean of the five independent biological replicates with five technical replicates. Data analysis for each graph is found on the figure legend.

## Data Availability

• All data reported in this paper will be shared by lead contact upon request. • This paper does not report original code. • Any additional information required to reanalyze the data reported in this paper is available from the lead contact upon request.
