# Supplementary material for: A polysaccharide deacetylase enhances bacterial adhesion in high-ionic-strength environments
Source: iScience. 2021 Sep 2;24(9):103071. doi: 10.1016/j.isci.2021.103071 (PMC8449245; doi:10.1016/j.isci.2021.103071)
Supplement: Document S1. Figures S1–S5 and Tables S1, S2 [file mmc1.pdf]

iScience, Volume *24*

Supplemental information

A polysaccharide deacetylase enhances bacterial adhesion in high-ionic-strength environments

Nelson K. Chepkwony and Yves V. Brun

Supplemental information

TABLE S1: Bacterial strains and plasmids, related to STAR Methods

Strain or Plasmid	Description and or genotype	Reference or source
E. coli		
α select	F^{-}*deoR endA1 relA1 gyrA96 hsdR17*($r_K^{-}m_K^{+}$) *supE44 thi-1 Δ(lacZYA-argFV169) Φ80δlacZ*ΔM15 λ	Bioline
BL21(DE3)	F^{-} *ompT hsdSB (rB-mB-) gal dcm (λDE3)*	New England Biolabs
YB8435	α select /pNPTS139Δ*hfsH*	This study
YB8432	α select /pNPTS139Δ*hfaB*	{Chepkwony, 2019 #75}
YB8428	α select/ pMR10::P*cu*-*hfsH*$_{HB}$	This study
YB9329	α select/ pMR10::P*cu*-*hfsH*$_{CC}$	This study
YB9328	BL21/ pET28a:*hfsH*$_{CC}$-*His*	This study
YB9327	BL21/ pET28a:*hfsH*$_{HB}$-*His*	This study
YB8444	α select/ pMR10::P*hfsE*$_{CC}$-*hfsH*$_{HB}$	This study
YB8443	α select/ pMR10::P*hfsE*$_{HB}$-*hfsH*$_{CC}$	This study
YB9535	α select/ pMR10::P*hfsE*$_{HB}$-*hfsH*$_{HB}$	This study
YB217	α select/ pMR10::P*xyl*$_{CC}$-*hfsH*$_{CC}$	This study
YB9536	α select/ pMR10::P*xyl*$_{CC}$-*hfsH*$_{HB}$	This study
YB9537	α select/ pMR10::P*xyl*$_{HB}$-*hfsH*$_{CC}$	This study
YB214	α select/ pMR10::P*xyl*$_{HB}$-*hfsH*$_{HB}$	This study
C. crescentus		
YB135	WT strain CB15	{Poindexter, 1964 #60}
YB9531	WT CB15 pMR10	This study
YB2198	CB15 Δ*hfsH*	{Toh, 2008 #68}
YB8662	CB15 Δ*hfsK*	{Sprecher, 2017 #65}
YB4251	CB15 Δ*hfaB*	{Hardy, 2010 #32}
YB9532	CB15 Δ*hfaB* pMR10	This study
YB9540	CB15 Δ*hfsH* pMR10::P*hfsE*$_{CC}$-*hfsH*$_{CC}$	{Toh, 2008 #68}
YB9534	CB15 Δ*hfsH* pMR10::P*hfsE*$_{CC}$-*hfsH*$_{HB}$	This study
YB6887	CB15 Δ*hfsH* pMR10::P*xyl*$_{CC}$-*hfsH*$_{CC}$	{Wan, 2013 #71}
YB221	CB15 Δ*hfsH* pMR10::P*xyl*$_{CC}$-*hfsH*$_{HB}$	This study
YB9627	CB15 Δ*hfsH* pMR10::P*xyl*$_{CC}$-*hfsH*$_{HB}^{D43A}$	This study
YB9533	CB15 Δ*hfaB* Δ*hfsH*	This study
YB9538	CB15 Δ*hfaB* Δ*hfsH* pMR10::P*xyl*$_{CC}$-*hfsH*$_{CC}$	This study
YB223	CB15 Δ*hfaB* Δ*hfsH* pMR10::P*xyl*$_{CC}$-*hfsH*$_{HB}$	This study
H. baltica		
YB5842	WT strain	{Schlesner, 1990 #61}
YB8438	WT pMR10	{Chepkwony, 2019 #75}
YB8404	YB5842 Δ*hfsA*	{Chepkwony, 2019 #75}
YB8415	YB5842 Δ*hfsH*	This study
YB9326	YB5842 D43A*hfsH*	This study
YB8406	YB5842 Δ*hfaB*	{Chepkwony, 2019 #75}
YB8412	YB5842 Δ*hbal_1607*	This study
YB8419	YB5842 Δ*hbal_hfsK*	This study
YB9541	YB5842 Δ*hbal_1184*	This study
YB9542	YB5842 Δ*hfsK* Δ*hbal_1607*	This study
YB9543	YB5842 Δ*hfsK* Δ*hbal_1184*	This study
YB9544	YB5842 Δ*hfsK* Δ*hbal_1184* Δ*hbal_1607*	This study
YB8417	YB5842 Δ*hfaB* pMR10	{Chepkwony, 2019 #75}
YB8416	YB5842 Δ*hfaB* Δ*hfsH*	This study
YB9318	YB5842 Δ*hfsH* pMR10::P*cu*-*hfsH*$_{CC}$	This study
YB8422	YB5842 Δ*hfsH* pMR10::P*cu*-*hfsH*$_{HB}$	This study
YB8423	YB5842 Δ*hfsH* pMR10::P*hfsE*$_{HB}$-*hfsH*$_{CC}$	This study
YB8421	YB5842 Δ*hfsH* pMR10::P*hfsE*$_{HB}$-*hfsH*$_{HB}$	This study
YB8420	YB5842 Δ*hfsH* pMR10::P*xyl*$_{HB}$-*hfsH*$_{CC}$	This study
YB218	YB5842 Δ*hfsH* pMR10::P*xyl*$_{HB}$-*hfsH*$_{HB}$	This study
YB222	YB5842 Δ*hfaB* Δ*hfsH* pMR10::P*xyl*$_{HB}$-*hfsH*$_{CC}$	This study
YB219	YB5842 Δ*hfaB* Δ*hfsH* pMR10::P*xyl*$_{HB}$-*hfsH*$_{HB}$	This study
YB9539	YB5842 Δ*hfaB* Δ*hfsH* pMR10::P*cu*-*hfsH*$_{CC}$	This study
YB185	YB5842 Δ*hfaB* Δ*hfsH* pMR10::P*cu*-*hfsH*$_{HB}$	This study
YB9332	YB5842 Δ*hfsH* pMR10::P*cu*-*hfsH*$_{HB}$ -X3FLAG	This study
YB9331	YB5842 Δ*hfsH* pMR10::P*cu*-*hfsH*$_{CC}$ -X3FLAG	This study
**Plasmids**		
pET28a(+)	Vector carrying an N- and C-terminal His-tag/ thrombin/T7-tag for protein overexpression	Novagen
pET28a *hfsH*$_{CC}$	Protein overexpression vector that carries the *hfsH*$_{CC}$	This study
pET28a *hfsH*$_{HB}$	Protein overexpression vector that carries the *hfsH*$_{HB}$	This study
pMR10	Mini-RK2 cloning vector; RK2 replication and stabilization functions	R. Roberts and C. Mohr
pMR10::P*cu*-*hfsH*$_{HB}$	pMR10 containing *hfsH*$_{HB}$ under copper inducible promoter	This study
pMR10::P*cu*-*hfsH*$_{CC}$	pMR10 containing *hfsH*$_{CC}$ under copper inducible promoter	This study
pMR10::P*hfsE*$_{CC}$-*hfsH*$_{CC}$	Complementation vector that carries *hfsH*$_{CC}$ under its native promoter	This study
pMR10::P*hfsE*$_{HB}$-*hfsH*$_{CC}$	Complementation vector that carries *hfsH*$_{CC}$ under its *H. baltica* native promoter	This study
pMR10::P*hfsE*$_{CC}$-*hfsH*$_{HB}$	Complementation vector that carries *hfsH*$_{HB}$ under its *C. crescentus* native promoter	This study
pMR10::P*hfsE*$_{HB}$-*hfsH*$_{HB}$	Complementation vector that carries *hfsH*$_{HB}$ under its native promoter	This study
pMR10::P*cu*-*hfsH*$_{HB}$ -X3FLAG	Triple FLAG tagged $HfsH_{HB}$	This study
pMR10::P*cu*-*hfsH*$_{CC}$ -X3FLAG	Triple FLAG tagged $HfsH_{CC}$	This study
pNPTS139	pLitmus 39 derivative, *ori*T, *sac*B, Kan^{r}	M.R..K Alley
pNPTS139Δ*hfsH*	pNPTS139 containing 500 bp fragments upstream and downstream of *hfsH*	This study
pNPTS139Δ*hfaB*	pNPTS139 containing 500 bp fragments upstream and downstream of *hfaB*	{Chepkwony, 2019 #75}

Tables S2: Primers, related to STAR Methods

Primers	Sequence (5'→3')	Description
hbal*hfsH*pupF	GCCAAGCTTCTCTGCAGGATGGGCCCCAGAATATGCTTTGCATCGG	5' region for deletion of *hfsH*
hbal*hfsH*upR	CAATTCTTCGACGATCCCAGTGTTGCTCACATCAACTGGC	
hbal*hfsH*dwF	GCCAGTTGATGTGAGCAACACTGGGATCGTCGAAGAATTG	3' region for deletion of *hfsH*
hbal*hfsH*pdwR	GCGAATTCGTGGATCCAGATCATCAAGGAAAGCATTTCCACAGCCAAAAG	
P*hfsE*$_{HB}$_*hfsH*$_{HB}$upF	CCATGATTACGCCAAGCTTCCATGGGATGGCCATACAAATATAAGCGGTGCTC	Complementation of *hfsH* using P*hfs* promoter from *H. baltica*
P*hfsE*$_{HB}$_*hfsH*$_{HB}$upR	GAGGGTGTGTAATGCCAATCAATCATTCACGAAGAACACAGAGTGTCTC	
P*hfsE*$_{HB}$_*hfsH*$_{HB}$dwF	GAGACACTCTGTGTTCTTCGTGAATGATTGATTGGCATTACACACCCTC	
P*hfsE*$_{HB}$_*hfsH*$_{HB}$dwR	CTAGAGCTCTGCAGGAGATCTCGATTCATGAGATTAGGCCGTAGCTTCTTG	
P*cu*_*hfsH*$_{HB}$upF	CCATGATTACGCCAAGCTTCCATGGGATTATACACGGATCGCACGCCTG	Complementation of *hfsH*$_{HB}$ using copper promoter P*cu* from *H. baltica*
P*cu*_*hfsH*$_{HB}$upR	GGGTGTGTAATGCCAATCAATCATGATGTTCTCCTTCTTGCGTTGGAC	
P*cu*_*hfsH*$_{HB}$dwF	GTCCAACGCAAGAAGGAGAACATCATGATTGATTGGCATTACACACCC	
P*cu*_*hfsH*$_{HB}$dwR	CTAGAGCTCTGCAGGAGATCTCGATAAAGCAGCATCCGTCATATTCAGAATGC	
P*hfsE*$_{HB}$_*hfsH*$_{CC}$upF	CCATGATTACGCCAAGCTTCCATGGGATGGCCATACAAATATAAGCGGTGCTC	Cross complementation of *hfsH*$_{CC}$ using *H. baltica* promoter
P*hfsE*$_{HB}$_*hfsH*$_{CC}$upR	CCGCTGCTGTGATGATGATGATGATGCATTCACGAAGAACACAGAGTGTCTC	
P*hfsE*$_{HB}$_*hfsH*$_{CC}$dwF	GAGACACTCTGTGTTCTTCGTGAATGCATCATCATCATCATCACAGCAGCGG	
P*hfsE*$_{HB}$_*hfsH*$_{CC}$dwR	CCATGATTACGCCAAGCTTCCATGGGATTTAGAGCCCGATCCGCCGCGAGCC	
P*cu*_*hfsH*$_{CC}$upF	CTAGAGCTCTGCAGGAGATCTCGATGGAAAGCGACAATAATCCAGTGTTCGAG	Cross complementation of *hfsH*$_{CC}$ using *H. baltica* copper promoter
P*cu*_*hfsH*$_{CC}$upR	GACCTTCTCGAATTCCATCGGCATCCCCCACATGCAGATTGCCATTGTC	
P*cu*_*hfsH*$_{CC}$dwF	GACAATGGCAATCTGCATGTGGGGGATGCCGATGGAATTCGAGAAGGTC	
P*cu*_*hfsH*$_{CC}$dwR	CCATGATTACGCCAAGCTTCCATGGGATCAGTGGTGGTGGTGGTGGTGCTCG	
P*xly*$_{HB}$_*hfsH*$_{HB}$upF	CCATGATTACGCCAAGCTTCCATGGGATGCTCGGAAACAGCCGCCAATAC	Complementation of *hfsH*$_{HB}$ using xylose promoter from *H. baltica* for overexpression
P*xly*$_{HB}$_*hfsH*$_{HB}$upR	GGGTGTGTAATGCCAATCAATCATTATATCAATTCACTCCACTATTTCATG	
P*xly*$_{HB}$_*hfsH*$_{HB}$dwF	CATGAAATAGTGGAGTGAATTGATATAATGATTGATTGGCATTACACACCC	
P*xly*$_{HB}$_*hfsH*$_{HB}$dwR	CTAGAGCTCTGCAGGAGATCTCGATAAAGCAGCATCCGTCATATTCAGAATGC	
P*xly*$_{CC}$_*hfsH*$_{CC}$upF	CCATGATTACGCCAAGCTTCCATGGGATACCAGCCACAGGCCCGTGCCGGGAT	Complementation of *hfsH*$_{CC}$ using xylose promoter from *C. crescentus* for overexpression
P*xly*$_{CC}$_*hfsH*$_{CC}$upR	GCTGCTGTGATGATGATGATGATGCATGGCGTCGTCTCCCCAAAACTCGAGCGTCT	
P*xly*$_{CC}$_*hfsH*$_{CC}$dwF	GACGCTCGAGTTTTGGGGAGACGACGCCATGCATCATCATCATCATCACAGCAGC	
P*xly*$_{CC}$_*hfsH*$_{CC}$dwR	ATGATTACGCCAAGCTTCCATGGGATTTAGAGCCCGATCCGCCGCGAGCC	
P*xly*$_{HB}$_*hfsH*$_{CC}$upF	CCATGATTACGCCAAGCTTCCATGGGATGCTCGGAAACAGCCGCCAATAC	Cross complementation of *hfsH*$_{CC}$ using *H. baltica xylose* promoter for overexpression
P*xly*$_{HB}$_*hfsH*$_{CC}$upR	CCGCTGCTGTGATGATGATGATGATGCATTATATCAATTCACTCCACTATTTCATG	
P*xly*$_{HB}$_*hfsH*$_{CC}$dwF	CATGAAATAGTGGAGTGAATTGATATAATGCATCATCATCATCATCACAGCAGC	
P*xly*$_{HB}$_*hfsH*$_{CC}$dwR	CCATGATTACGCCAAGCTTCCATGGGATTTAGAGCCCGATCCGCCGCGAGCC	
P*xly*$_{CC}$_*hfsH*$_{HB}$upF	CCATGATTACGCCAAGCTTCCATGGGATACCAGCCACAGGCCCGTGCCGGGATC	Cross complementation of *hfsH*$_{HB}$ using *C. crescentus xylose* promoter for overexpression
P*xly*$_{CC}$_*hfsH*$_{HB}$upR	GGGTGTGTAATGCCAATCAATCATGGCGTCGTCTCCCCAAAACTCGAGCGTCTG	
P*xly*$_{CC}$_*hfsH*$_{HB}$dwF	CAGACGCTCGAGTTTTGGGGAGACGACGCCATGATTGATTGGCATTACACACCC	
P*xly*$_{CC}$_*hfsH*$_{HB}$dwR	CTAGAGCTCTGCAGGAGATCTCGATAAAGCAGCATCCGTCATATTCAGAATGC	
hbal*hfsK*pupF	GTGCTAGCGAATTCTGGATCCACGATATTCAATTTCATCAATGAAGATTGGGC	5' region for deletion of *hfsK*
hbal*hfsK*upR	CAGAAATATTTTGCTTCTGGTTTACAGAATGCTCTTCTAACTTCGCAAAC	
hbal*hfsK*dwF	GTTTGCGAAGTTAGAAGAGCATTCTGTAAACCAGAAGCAAAATATTTCTG	3' region for deletion of *hfsK*
hbal*hfsK*pdwR	GGCGCCAGAAAGCTTCCTGCAGGATCTCAACCTTCACAAAGGGAGTTGATTTG	
hbal*1184*pupF	GCCAAGCTTCTCTGCAGGATATGCAGTTTGAAGTTGTGTCTCCAGAAG	5' region for deletion of *hbal_1184*
hbal*1184*upR	CTTAAAAAAATGATCCCAACCGACAAGTCATACCCTACAGACAAATC	
hbal*1184*dwF	GATTTGTCTGTAGGGTATGACTTGTCGGTTGGGATCATTTTTTTAAG	3' region for deletion of *hbal_1184*
hbal*1184*pdwR	GCGAATTCGTGGATCCAGATCGCTGTTTCAATCGCTCTTGGTCG	
hbal*1607*pupF	GCCAAGCTTCTCTGCAGGATGTGGTTTTATTTGTTGCAAAAGATATTTC	5' region for deletion of *hbal_1607*
hbal*1607*upR	GAGGTGAGAGATACAAAATCTTCCGACTAAGCCGCCGAAAG	
hbal*1607*dwF	CTTTCGGCGGCTTAGTCGGAAGATTTTGTATCTCTCACCTC	3' region for deletion of *hbal_1607*
hbal*1607*pdwR	GCGAATTCGTGGATCCAGATGTGATCTCAATCCAACTATCTG	
FlgHfsH$_{HB}$UpF	GCGCCTTAATTAATATGCATGGTACATGATTGATTGGCATTACACACCCTC	Tagging HfsH$_{HB}$ with 3XFLAG epitope tag
FlghfsH$_{HB}$dwR	CCGGAGCTCGAGATCTTAAGGTACCTGAGATTAGGCCGTAGCTTCTTGCTG	
P*cu*_HfsH$_{HB}$UpR	GAGGGTGTGTAATGCCAATCAATCATGATGTTCTCCTTCTTGCGTTGGACG	
P*cu*HfsH$_{HB}$DwF	CGTCCAACGCAAGAAGGAGAACATCATGATTGATTGGCATTACACACCCTC	
flgHfsH$_{CC}$upF	GCGCCTTAATTAATATGCATGGTACATGCCGATGGAATTCGAGAAGGTCG	Tagging HfsH$_{CC}$ with 3XFLAG epitope tag
flgHfsH$_{CC}$dwR	CCGGAGCTCGAGATCTTAAGGTACCGAGCCCGATCCGCCGCGAGCC	
P*cu*_hfsH$_{CC}$UpR	CGACCTTCTCGAATTCCATCGGCATGATGTTCTCCTTCTTGCGTTGGACG	
P*cu*HfsH$_{CC}$DwF	CGTCCAACGCAAGAAGGAGAACATCATGCCGATGGAATTCGAGAAGGTCG	

SUPPORTING FIGURES AND FIGURE LEGENDS

Figure S1. The HfsK acetyltransferase is not required for holdfast adhesion and biofilm formation in *H. baltica,* related to Figure 2

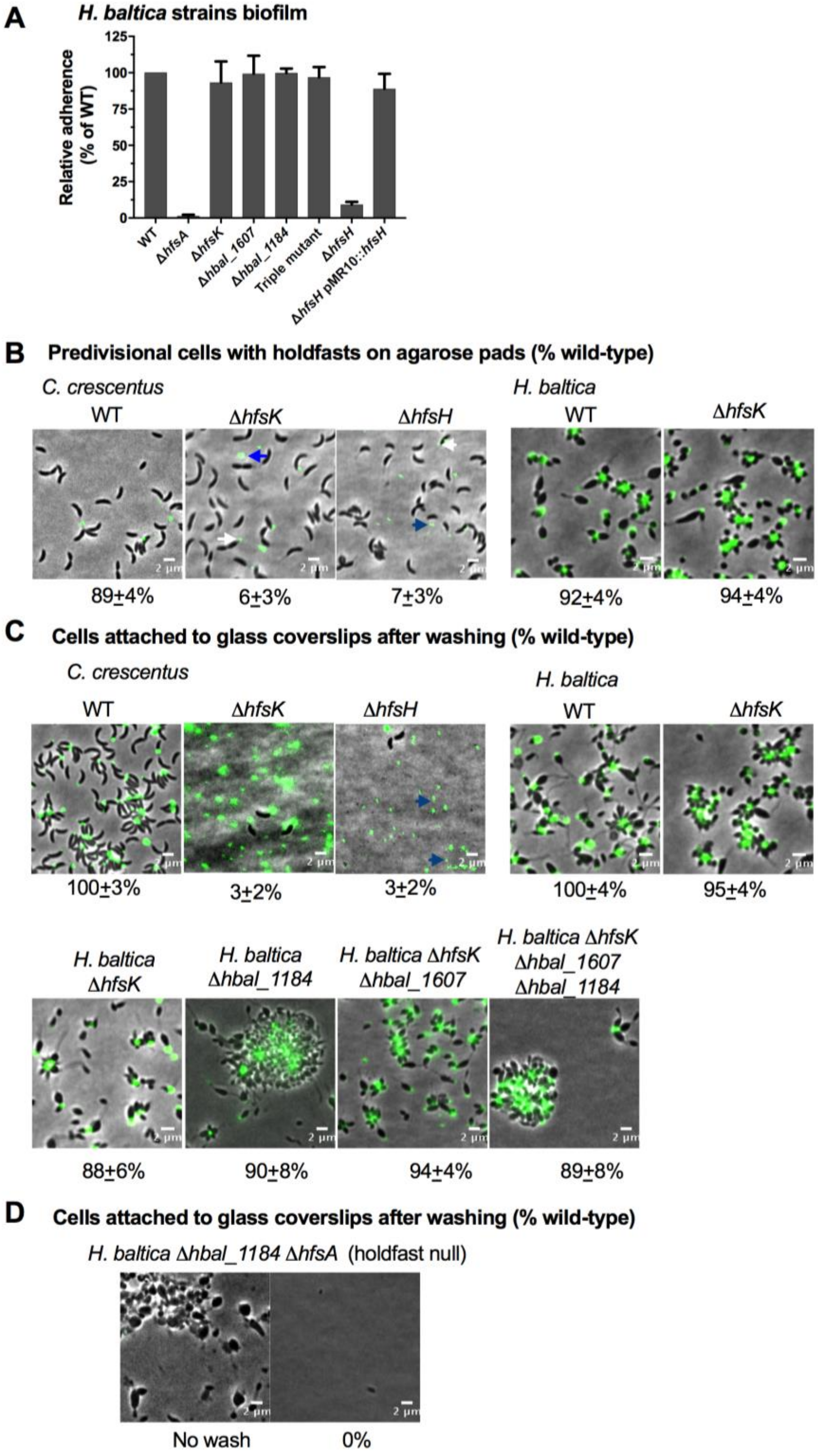

Figure S1. The HfsK acetyltransferase is not required for holdfast adhesion and biofilm formation in *H. baltica,* related to Figure 2

A. Quantification of biofilm formation by the crystal violet assay after incubation for 12 h, expressed as a mean percent of WT crystal violet staining. Holdfast null strain Δ*hfsA* was used as a negative control. Error is expressed as the standard error of the mean of three independent biological replicates with four technical replicates each. The triple mutant is *H. baltica* Δ*hfsK* Δ*hbal_1607* Δ*hbal_1184.* **B.** Representative images showing merged phase and fluorescence channels of the indicated *C. crescentus* and *H. baltica* strains on agarose pads. Holdfast is labeled with AF488-WGA (green). White arrows indicate holdfasts attached to the Δ*hfsH* and Δ*hfsK* cells, and blue arrows indicate holdfast shed into the medium. Exponential planktonic cultures were used to quantify the percentage of predivisional cells with holdfast. Data are expressed as the mean of three independent biological replicates with four technical replicates each. Error bars represent the standard error of the mean. A total of 3,000 cells were quantified per replicate using MicrobeJ (Images for the WT and *hfsH* are from Fig. 2). **C-D.** Representative images showing merged phase and fluorescence channels of *C. crescentus* and *H. baltica* strains bound to a glass coverslip. Exponential cultures were incubated on the glass slides for 1 h, washed to remove unbound cells, and holdfast were labelled with AF488-WGA (green). Blue arrows indicate surface-bound holdfasts shed by *hfsH* mutants. The data showing quantification of cells bound to the glass coverslip are the mean of two biological replicates with five technical replicates each. Error is expressed as the standard error of the mean using MicrobeJ (Images for the WT and *hfsH* are from Fig. 2).

Figure S2. ***H. baltica*** **and** ***C. crescentus*** **holdfast modification enzymes HfsK and HfsH, related to Figure 2*.***

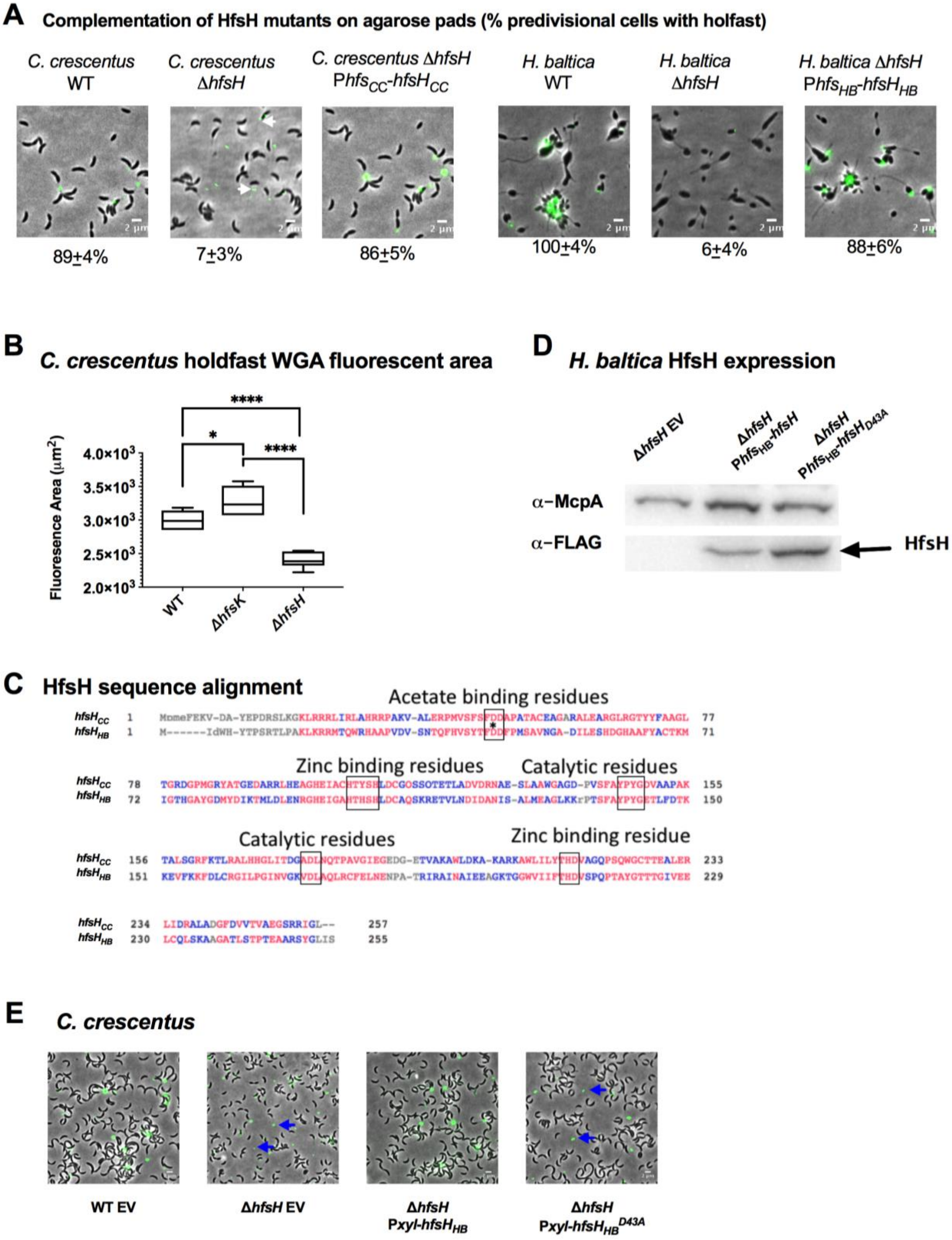

Figure S2. *H. baltica* and *C. crescentus* modification enzymes HfsK and HfsH, related to Figure 2.

A. Images showing merged phase and fluorescence channels of the indicated strains of *C. crescentus* (left) and *H. baltica* (right). Holdfast is labeled with AF488-WGA (green). Exponential planktonic cultures were used to quantify the percentage of predivisional cells with holdfast. Data is expressed as the mean of three independent biological replicates with four technical replicates each. Error bars represent the standard error of the mean. **B.** Box plot showing the area of AF488-WGA fluorescence from holdfast produced by *C. crescentus* strains. Data is the mean of four biological replicates. The horizontal bar represents the median, the box represents 25th and 75th percentile, and the whiskers represent the full range of data. * and *** represent *P values* <0.1 and <0.0001 **C.** Alignment of the *C. crescentus* and *H. baltica* HfsH amino acid sequences with conserved carbohydrate esterase family 4 (CE4) motifs indicated by rectangles. The conserved residue involved in acetate binding is indicated with an asterisk (D48 in *C. crescentus* HfsH and D43 in *H. baltica* HfsH). **D.** Western blots of whole cell lysates of the indicated strains showing the expression level of FLAG-tag fusions of HfsH and HfsH^{D43A}. McpA levels were monitored as a loading control. **E**. Images showing merged phase and fluorescence channels of the indicated strains of *C. crescentus* and *C. crescentus* Δ*hfsH* cross-complemented with *hfsH* from *H. baltica*: $hfsH_{HB}$ (WT) and $hfsH_{HB}^{D43A}$ (active site mutant). Holdfast is labeled with AF488-WGA (green) and blue arrows indicate shed holdfasts.

Figure S3. HfsH expression using a copper inducible promoter (P_{Cu}) in *H. baltica,* related to Figure 4

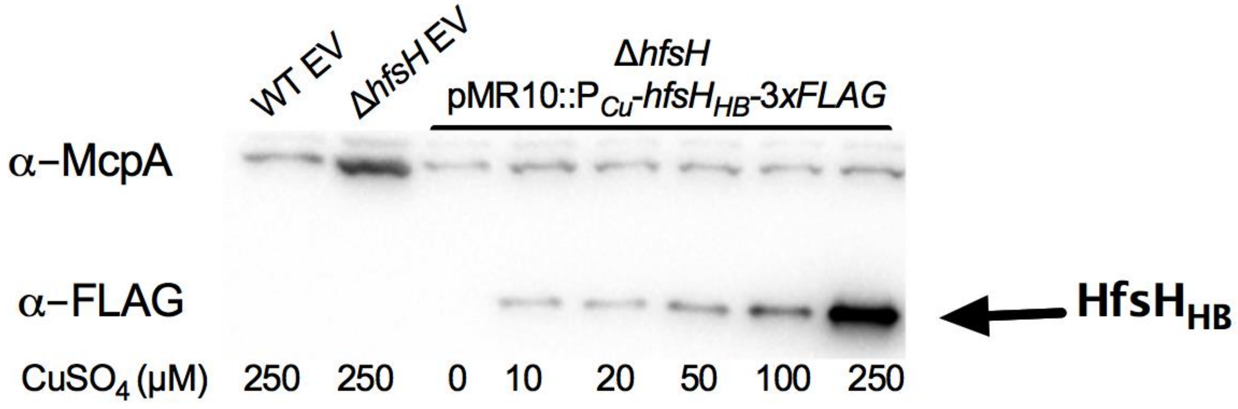

Figure S3. HfsH expression using a copper inducible promoter (P_{Cu}) in *H. baltica,* related to Figure 4

Western blots of whole cell lysates showing the expression levels of FLAG-tagged HfsH under the control of the copper inducible promoter after 4 h of induction with 0 – 250 µM $CuSO_4$. McpA levels were monitored as a loading control.

Figure S4. Levels of $HfsH_{HB}$ and $HfsH_{CC}$ expression using P*hfs* and P*xyl* promoters in *H. baltica,* related to Figure 5

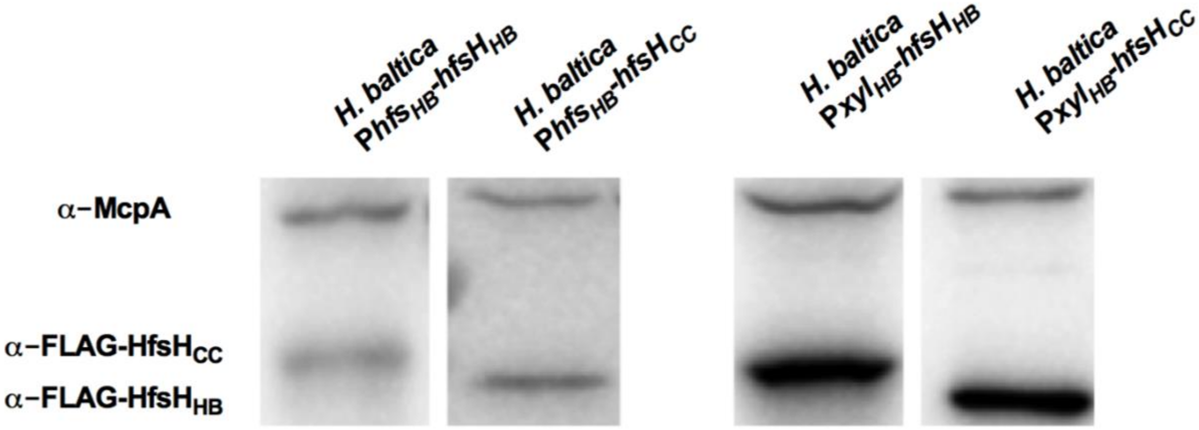

Figure S4. Levels of $HfsH_{HB}$ and $HfsH_{CC}$ expression using P*hfs* and P*xyl* promoters in *H. baltica,* related to Figure 5

Western blots of whole cell lysates showing the expression levels of FLAG-tagged $HfsH_{HB}$ and $HfsH_{CC}$ under the control of native holdfast synthesis (P*hfs)* and xylose-inducible (P*xyl*) promoters after 4 h of induction with 0.03% xylose. McpA levels were monitored as a loading control.

Figure S5. Cross-complementation of $HfsH_{HB}$ and $HfsH_{CC}$ using the copper inducible promoter, related to Figure 5

A WGA fluorescent intensity of holdfast decetylated by $HfsH_{HB}$ and $HfsH_{CC}$ in *H. baltica* with 50 µM $CuSO_4$

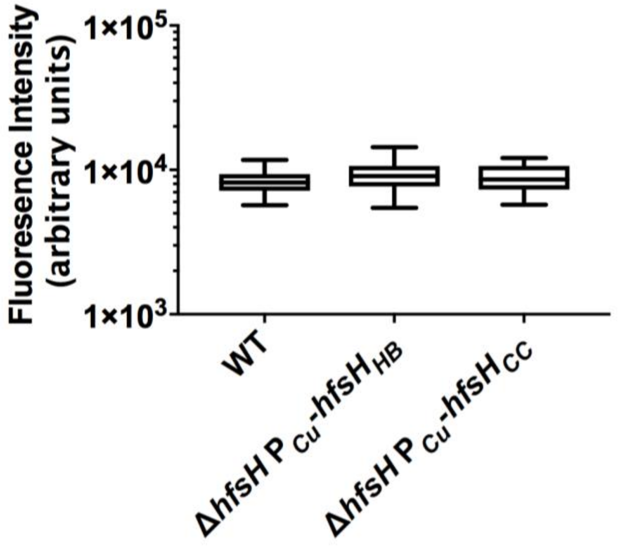

B $HfsH_{CC}$ and $HfsH_{HB}$ expression using a copper inducible promoter

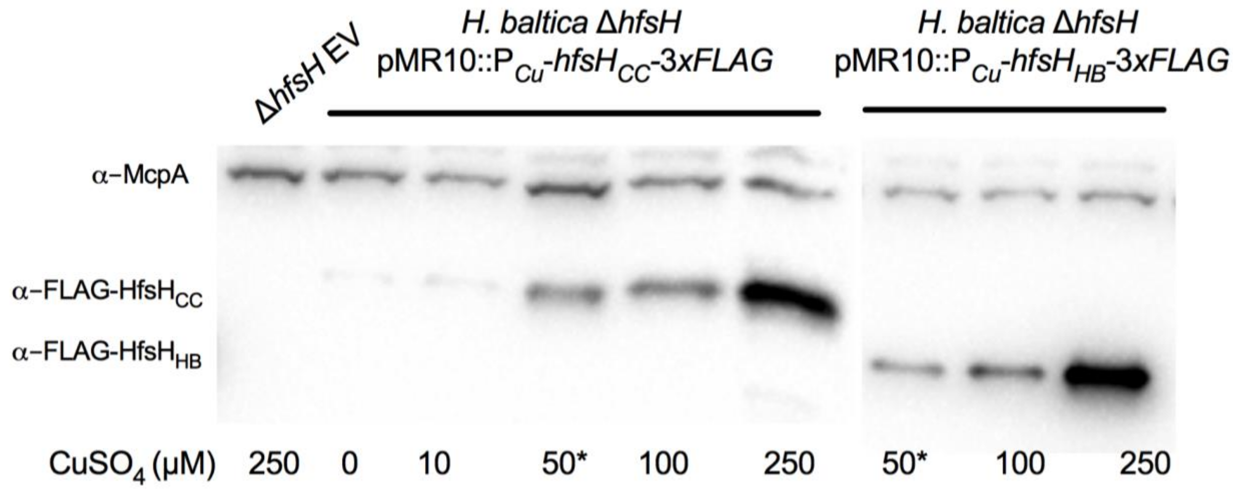

Figure S5. Cross-complementation of $HfsH_{HB}$ and $HfsH_{CC}$ using the copper inducible promoter, related to Figure 5

A. Box plot showing the integrated intensity of AF488-WGA fluorescence from holdfast produced by *H. baltica* Δ*hfsH* pMR10::P_{Cu}-*hfsH*$_{HB}$ and cross-complemented *H. baltica* Δ*hfsH* pMR10::P_{Cu}-*hfsH*$_{CC}$ at 50 µM $CuSO_4$ induction for 4 h. Data is the mean of four biological replicates. The horizontal bar represents the median, the box represents 25th and 75th percentile, and the whiskers represent the full range of data. **B.** Western blots of whole cell lysates showing the expression level of FLAG-tagged $HfsH_{HB}$ and $HfsH_{CC}$ under the control of the copper inducible promoter after 4 h of induction with 0 – 250 µM $CuSO_4$. McpA levels were monitored as a loading control. The star indicates HfsH induction at 50 µM $CuSO_4$, used in comparing *H. baltica* and *C. crescentus* holdfast binding.
